# Pilin antigenic variants impact gonococcal lifestyle and antibiotic tolerance by modulating interbacterial forces

**DOI:** 10.1371/journal.pbio.3003022

**Published:** 2025-01-30

**Authors:** Isabelle Wielert, Sebastian Kraus-Römer, Thorsten E. Volkmann, Lisa Craig, Paul G. Higgins, Berenike Maier

**Affiliations:** 1 Institute for Biological Physics, University of Cologne, Cologne, Germany; 2 Center for Molecular Medicine Cologne, Cologne, Germany; 3 Department of Molecular Biology and Biochemistry, Simon Fraser University, Burnaby, British Columbia, Canada; 4 Institute for Medical Microbiology, Immunology and Hygiene, Faculty of Medicine and University Hospital Cologne, University of Cologne, Cologne, Germany; 5 German Centre for Infection Research, Partner site Bonn-Cologne, Cologne, Germany; Max Planck Institute for Terrestrial Microbiology: Max-Planck-Institut fur terrestrische Mikrobiologie, GERMANY

## Abstract

Type 4 pili (T4P) are multifunctional filaments involved in adhesion, surface motility, biofilm formation, and horizontal gene transfer. These extracellular polymers are surface-exposed and, therefore, act as antigens. The human pathogen *Neisseria gonorrhoeae* uses pilin antigenic variation to escape immune surveillance, yet it is unclear how antigenic variation impacts most other functions of T4P. Here, we addressed this question by replacing the major pilin of a laboratory strain with pilins from clinical isolates. We reveal that the resulting strains vary substantially in their attractive forces. Strongly interacting bacteria form microcolonies while weakly interacting bacteria retain a planktonic lifestyle. In mixed microcolonies, different variant strains segregate in agreement with the differential strength of adhesion hypothesis. By combining structural predictions and laser tweezers experiments, we show that the C-terminal region of the pilin is crucial for attraction. Lifestyle affects growth kinetics and antibiotic tolerance. In the presence of ceftriaxone or ciprofloxacin, the killing kinetics indicate strongly increased tolerance of aggregating strains. We propose that pilin antigenic variation produces a mixed population containing variants optimized for growth, colonization, or survivability under external stress. Different environments select different variants, ensuring the survival and reproduction of the population as a whole.

## Abstract 2

Neisseria gonorrhoeae evades immunity by altering its surface structures through antigenic variation. This study shows that this process also modulates bacterial interactions, driving microcolony diversification, aggregation, colony morphology, and antibiotic tolerance.

## Introduction

Type 4 pili (T4P) are filamentous cell appendages generated by a variety of pathogenic bacteria including the *Neisseria* species, *Pseudomonas aeruginosa*, *Vibrio cholerae*, and *Acinetobacter baumannii*. They support functions such as adhesion, motility, aggregation, and horizontal gene transfer, which are crucial for survival, colonization, and virulence [1,2]. In pathogenic *Neisseria*, the amino acid sequence of the T4P major pilin, PilE, continually changes as a result of antigenic variation [3,4]. This role of PilE variability in immune escape is well-established [5–7] and it has been shown that it can affect adhesion to host cells [8]. Currently, its implications in other pilus-mediated functions and how these influence gonococcal planktonic versus biofilm lifestyle are not well understood.

T4P are polymers of the major pilin, with multiple low-abundance minor pilins [2]. For gram-negative bacteria, they protrude from a complex that spans the inner and outer membrane [2]. Driven by cytosolic ATPases, the pilus filament elongates by polymerization and retracts by depolymerization. In *N*. *gonorrhoeae*, the gene encoding the major pilin PilE is hypermutable by antigenic variation. Pilin antigenic variation was originally discovered by the observation that the morphology of gonococcal colonies on agar plates is related to piliation [3,6,7]. Piliated *N*. *gonorrhoeae* produce convex colonies, whereas non-piliated variants produce flat colonies. A nonpiliated phenotype is mainly caused by an antigenic variation that generates a truncated PilE, e.g., by a frameshift mutation [9,10]. In general, the macroscopic phenotype can be linked to the strength of interactions generated by the T4P of neighboring cells [11]. Antigenic variation occurs at a rate of 1.7∙10^−3^ events/cell/generation for the gonococcal lab strain MS11 [12]. Segments of one of the up to 18 different *pilS* sequences [13,14] recombine with *pilE* and replace extended stretches of the *pilE* sequence. The variability along the *pilE* sequence is heterogeneous and can be subdivided into conserved, semi-variable and hyper-variable regions [15]. Pilin antigenic variation relies on a G4 motif upstream of *pilE*; deletion of this motif abolishes antigenic variation [16]. Using a mutational screen of the 3′ region of the gonococcal *pilE* coding sequence, it was shown that various point mutations caused loss of piliation [10]. For closely related *N*. *meningitidis* T4P, mutational analysis of *pilE* was used to generate a functional map of the major pilin [17]. In that study, a specific major pilin variant was used as a reference structure and single amino acid changes in different regions were associated with different T4P functions including biogenesis, adhesion, and aggregation [17]. However, it remains elusive how naturally occurring antigenic variations, which extend along dozens of amino acid residues throughout the PilE sequence, affect T4P functionality.

In this study, we focus on the following T4P-related functions: twitching motility, generation of attractive forces between cells, microcolony formation, and antibiotic tolerance. Twitching motility is a mode of surface motility driven by cycles of T4P elongation, surface attachment, and retraction [2,18,19]. *N*. *gonorrhoeae* uses a tug-of-war mechanism for twitching motility [20,21] and, as a consequence, gonococcal motility can be described as a correlated random walk. While T4P surface attachment mediates motility, pilus:pilus binding between adjacent cells causes attraction between gonococci [22]. In liquid media, this attraction causes rapid aggregation of *Neisseria* species into spherical (micro)colonies comprising thousands of cells [22–24]. The strength of the attractive force determines whether the colonies behave as liquids or solids [22,25,26], which impacts tolerance to antibiotics [27]. Attractive forces are reduced by the posttranslational surface modification of the T4P [25]. If gonococcal strains generating different levels of attractive force are mixed, they can form colonies comprising both strains. The bacteria segregate [11,25] in agreement with the differential strength of adhesion hypothesis that states that the most strongly interacting cells accumulate at the centre of the colony [28]. So far it is unclear whether the PilE sequence and thus the pilus surface stereochemistry also affect the attractive forces and colony morphology. Here, we investigate how different variants of PilE impact the gonococcal lifestyle, i.e., whether they live as planktonic cells or within aggregates including microcolonies or biofilms.

The emergence of resistance to several classes of antibiotics has made *N*. *gonorrhoeae* a multidrug-resistant pathogen [29]. While the mechanisms conferring gonococcal drug resistance are fairly well understood [30,31], very little is known about antibiotic tolerance of this pathogen. Tolerance describes the ability of bacteria to survive antibiotic treatment for extended periods of time [32]. This extended survival time is problematic for eradication of the pathogen and often precedes antibiotic resistance [33]. The mechanisms driving tolerance are multifaceted including reduced permeability to antibiotics, reduction of growth rate and metabolic activity, membrane polarization, as well as the activation of stress responses [34]. A major tolerance mechanism is aggregation and biofilm formation [35]. Gonococcal aggregation enhances tolerance to ceftriaxone, a β-lactam antibiotic that targets cell wall synthesis [36] with the physical properties of the microcolony impacting the degree of tolerance [27]. Ceftriaxone is currently recommended for gonorrhea treatment. Since pilin antigenic variation potentially affects gonococcal aggregation and the physical properties of the colonies, it likely impacts antibiotic tolerance.

In this study, we investigate how variation of the pilin sequence affects gonococcal properties, behavior, and lifestyle. Based on the PilE sequences, we predict structural changes to the surface of the T4P filament that likely impact gonococcal aggregation. Using laser tweezers, we confirm that pilin variation strongly affects the attractive force between pairs of cells. We show that all *pilE* variants in this study produce functional T4P but cluster into 2 distinct phenotypic lifestyles; planktonic and aggregating, depending on the T4P-mediated attractive force. Structural predictions suggest that complementarity between knobs and cavities in adjacent pili influences cell-to-cell attraction. We confirm this prediction experimentally by generating strains expressing *pilE* hybrids between different variants. We show that pilus attractive forces and gonococcal aggregation, growth and survival under antibiotic treatment strongly depend on the pilin variant. Taken together, we reveal that some pilin antigenic variants generate aggregative T4P while others assemble into non-aggregative T4P with phenotypic consequences that optimize bacterial proliferation and survival under a variety of conditions including antibiotic treatment.

## Results

### Pilin antigenic variants show different stereochemistries of key interaction sites on T4P

**Fig 1:**
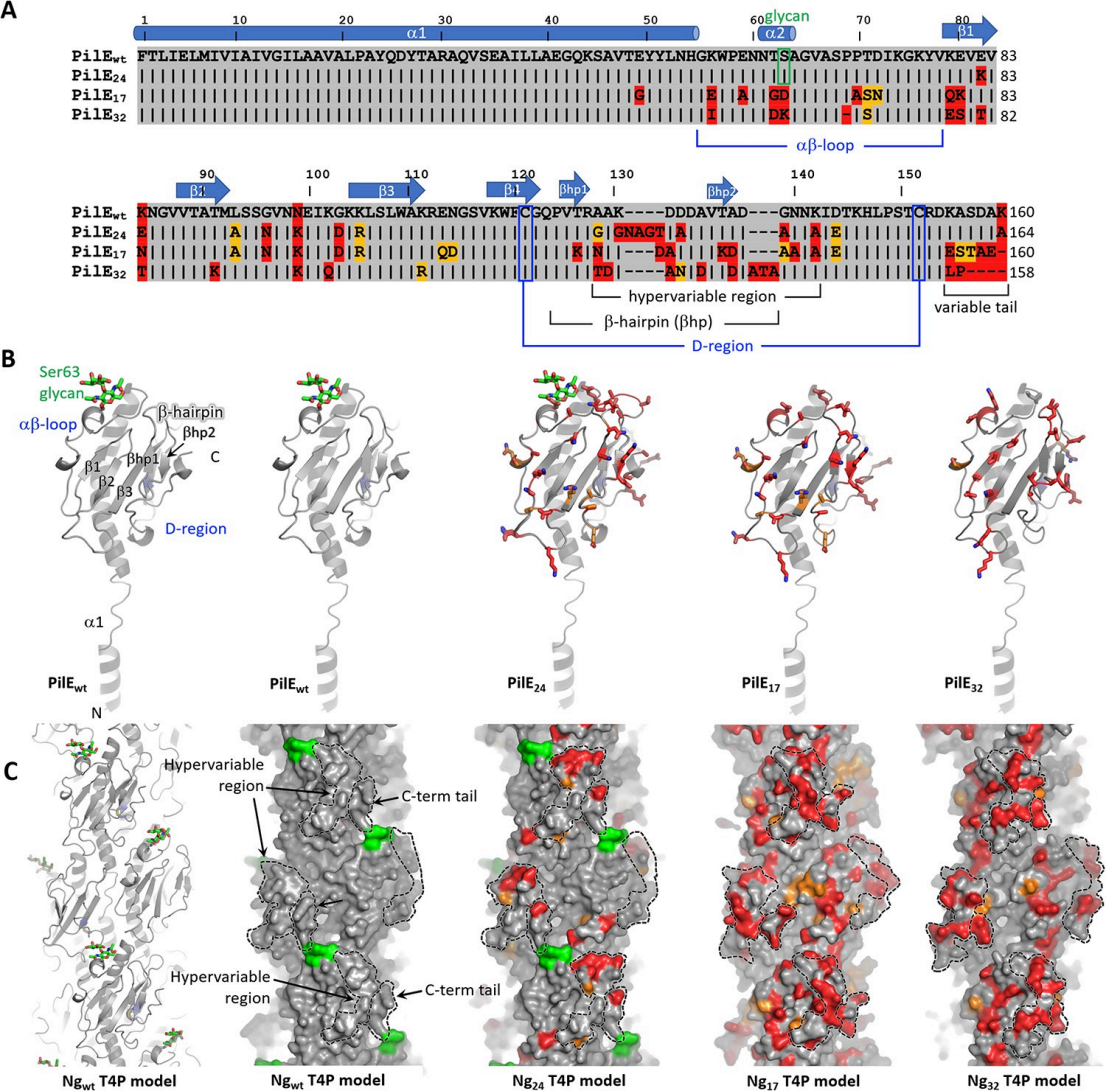
Sequence alignment and structure predictions for PilE variants and pilus filaments. (A) PilE amino acid sequence alignment for wt* and variant strains. Amino acids of the PilE variants that are identical to those in PilE_wt_ are shown as black bars with gray shading, conservative amino acid changes are indicated with orange shading and non-conserved residues have red shading. The secondary structure and other hallmark features of *Neisseria* Type 4 pilins are indicated based on alignment of PilE_wt_ with C30 PilE (Protein Data Bank ID 2HI2). (B) Pilin models were generated using AlphaFold [39,67]. Residues that differ from those in PilE_wt_ are shown in stick representation, with carbon colored as in Fig 1A, nitrogen in blue and oxygen in red. Glycan carbons are green. The conserved disulfide-bonded cysteines that delineate the D-region are blue. Variable residues are located on the face of the C-terminal globular domain. (C) Filament models were generated by superimposing the pilin predictions on the *N*. *gonorrhoeae* T4P structure (EMD-8739) and applying its symmetry parameters. The wt* T4P model is shown on the left in cartoon representation and all models are shown in surface representation, colored as in Fig 1B. The hypervariable β-hairpin and C-terminal tail together form a protruding “knob” on each subunit (dashed lines) and deep cavities or “holes” lie at the interface between subunits.

We sought to understand how pilin antigenic variation affects the pilus-mediated interactions between bacteria and to characterize the phenotypic changes including growth, antibiotic resistance, and antibiotic tolerance that result from altering these interactions. In the first step, we cloned the *pilE* genes from the *N*. *gonorrhoeae* clinical isolates Ng24, Ng17, and Ng32 into the background strain, wt*, in place of the native *pilE* gene. wt* is *N*. *gonorrhoeae* MS11 with the G4 motif deleted to prevent further antigenic variation of *pilE* [37]. The *pilS* sequences of the clinical isolates are similar to the respective sequences of the wt* strain (Fig i in S1 Text, S1 Data). Specific *pilS* sequences can be mapped nearly contiguously to the *pilE* sequences (Fig ii in S1 Text), indicating that the *pilE* variants of the clinical isolates have arisen by pilin antigenic variation. To understand the implications of differences in *pilE* sequences among variants, we examined their pilin sequences and predicted monomeric pilin structures and T4P filament structures. The amino acid sequences of the pilin variants were compared to that of the wt* strain (Fig 1A). Secondary structure and features were assigned based on alignment with the *N*. *gonorrhoeae* C30 PilE crystal structure [38], which is 90% identical in sequence to wt* PilE and 99% identical in the first 120 amino acids. The sequence of the N-terminal α-helix, α1, is identical for all pilins, with the exception of a glycine instead of glutamate at position 49 of Ng17 PilE (PilE_17_). PilE_wt_ and PilE_24_ share a serine at position 63, which is posttranslationally modified with a glycan in *N*. *gonorrhoeae* MS11 and C30 [38]. PilE_32_ and PilE_17_ have charged residues at this position. Some sequence variation is observed in the αβ-loop, which lies between α1 and strand β1 of the β-sheet, in β1 and in the β1-β2 loop. The most variable region is the hypervariable β-hairpin that follows the β-sheet and the C-terminal tail, which have amino acid changes but also insertions and deletions. We determined the overall pilin sequence identities of the variant to that of MS11 (Table ii in S1 Text). PilE_24_ is 90% identical to PilE_wt_, whereas PilE_32_ is 86.6% identical and PilE_17_ is 80.6% identical.

Pilin models for each variant were generated using AlphaFold [39]. The continuous N-terminal α-helix, α1, seen in the crystal structure of PilE [38] and in the AlphaFold predictions was replaced with the partially melted α-helix seen in the cryo-electron microscopy reconstruction of the intact pilus [40] (Fig 1B). All variable residues are located on the face of the PilE globular domain that is exposed in the pilus filament, as expected for antigenic variation. PilE_17_ shows the greatest degree of variability, particularly in the protruding β-hairpin and C-terminal tail.

Pilus filament models were generated by superimposing each pilin model onto a single subunit in the cryoEM structure of the *N*. *gonorrhoeae* T4P and applying its symmetry parameters (Fig 1C). Filament models are shown in Fig 1C. The T4P surface is undulating: the β-hairpin and the C-terminal tail regions, which show the highest sequence variability, together form protruding “knobs”; and deep cavities or grooves lie between the globular domains, lined with the variable C-terminal tail on one side and the αβ-loop and β1-β2 loop on the other. Accordingly, the conformation of both the knobs and the cavities/grooves (“holes”) vary considerably among the variants, with wt* and Ng24 T4P being most similar. The electrostatic surface potential also differs substantially from one pilus variant to the next (Fig iii in S1 Text), with positively charged residues framing the holes of PilE_wt_ and PilE_24_ and negatively charged residues lining the edges of the PilE_17_ and PilE_32_ holes. The knobs of each pilin differ in their distribution of charged residues, with PilE_32_ having the most negatively charged knob. Pilus:pilus interactions likely require both structural and chemical complementarity between these surface features to allow the knobs in one filament to fit into the holes in another. Thus, these marked differences in stereochemistry among the pilus variants could impact pilus:pilus interactions and bacterial aggregation.

### All PilE variants support twitching motility and high pilus densities

**Fig 2:**
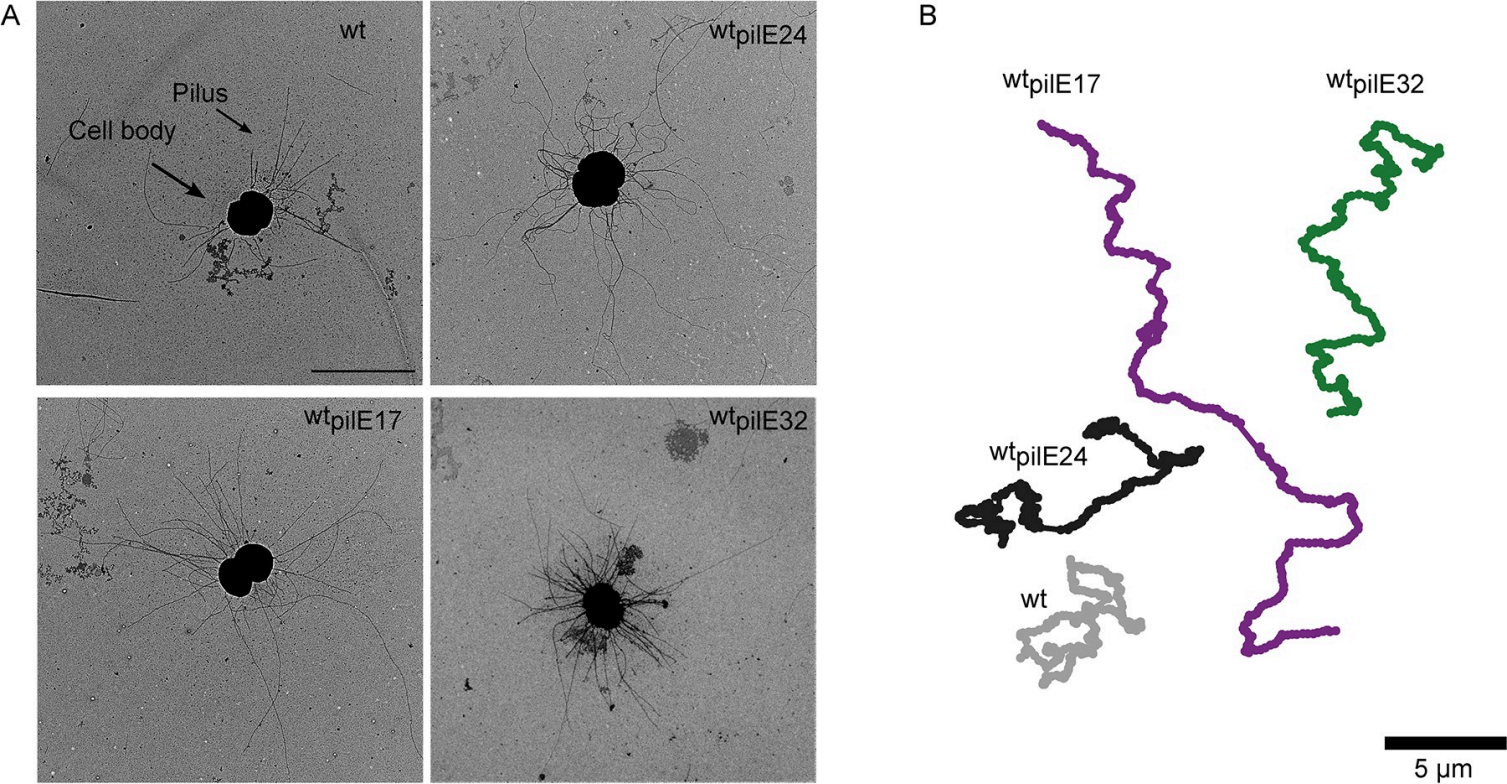
Replacing the native *pilE* sequence by antigenic variants does not reduce T4P density and maintains twitching motility. (A) Representative TEM images of pilin variants. Scale bar: 2 μm. (B) Trajectories of motile bacteria. Representative trajectories of pilin variants over 30 seconds (tracks made via Trackmate Image J [68]) (gray: wt* (Ng150), dark gray: wt_*pilE24*_ (Ng242), purple: wt_*pilE17*_ (Ng240), green: wt_*pilE32*_ (Ng230)). Scale bar: 5 μm.

Pilin antigenic variation can lead to loss of T4P function, in particular to reduced levels of piliation [9,10]. To test for piliation, we assessed the density of T4P for each strain using negative-stain transmission electron microscopy (TEM). All 4 strains show high levels of piliation (Fig 2A). Quantitatively, wt_*pilE32*_ has elevated number of pili per cell compared to the other strains which have comparable piliation levels (Fig iv in S1 Text).

**Fig 3:**
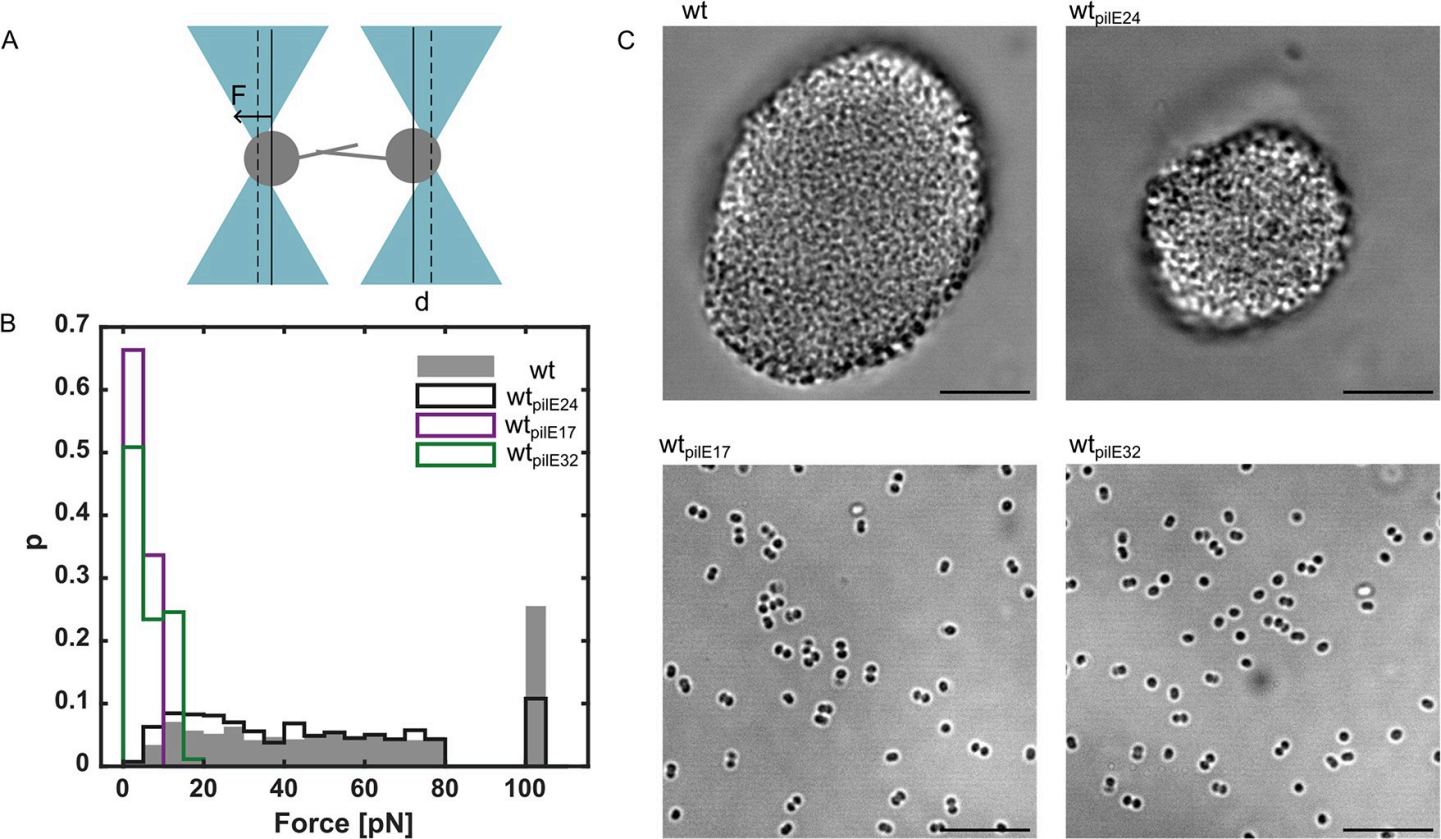
Attractive force between pairs of bacteria depends on the pilin sequence. (A) Sketch of dual laser tweezers setup. (B) Probability distribution of rupture forces (number of interacting cell pairs: N_wt_ = 71, N_wt*pilE24*_ = 59, N_wt*pilE17*_ = 30, N_wt*pilE32*_ = 54). The linearity of the laser trap is limited to 80 pN. All rupture events exceeding this force were grouped into a single bin shown at 100 pN. The data underlying this figure can be found in S1 Data. (C) Representative brightfield images of gonococci in liquid culture show aggregated microcolonies for wt and wt_pilE24_ and planktonic cells for wt_pilE17_ and wt_pilE32._ Scale bar: 10 μm.

We next compared T4P dynamics among the variants. T4P dynamics correlates quantitatively with twitching motility; the faster T4P retract the faster gonococci move on BSA-coated glass [20,21]. To find out whether variations in the PilE sequence affect T4P dynamics, we compared the trajectories of gonococci moving at the glass surface. All variants exhibit twitching motility on BSA-coated glass surfaces. A representative track for each *pilE* variant is shown in Fig 3B. A detailed analysis of the trajectories using a correlated random walk model [21] (described in Methods, Fig vi in S1 Text) showed that strain wt_*pilE17*_ is the most motile; both the velocity v_corr_ and the correlation time τ were significantly higher than for the wt* strain (Fig v in S1 Text). The correlation time is a measure of how long the bacterium moves without changing direction. We conclude that all PilE variants have comparable or even higher levels of pili and generate dynamic T4P.

### Replacing the native pilin in the laboratory strain with antigenic variants affects bacterial attractive forces and consequently aggregation and segregation

We examined the effects of the variations in pilus surface stereochemistry on pilus:pilus interaction and aggregation. In this study, the term pilus:pilus interaction is used to describe the interaction between T4P of adjacent gonococci. Using a dual laser tweezers assay, we investigated the attractive forces generated by the pilin variants. For each strain, we trapped pairs of bacteria and measured the T4P-mediated interaction forces between them (Fig 3A). When bacteria in different traps interact via their T4P, and at least one T4P retracts, the cell bodies approach each other. Bacteria are deflected by a distance *d* from the centers of the traps. As the deflection increases, the restoring force of the laser traps increases as well leading to a rupture event when the optical force exceeds the (rupture) force that the pilus:pilus bond can sustain.

The force at which a pilus:pilus bond ruptures was used as a measure of the attractive force between gonococci. *F*_*rupture*_ is defined as the maximal force attained before the bond breaks and the bacteria move back to their equilibrium positions. We did not observe significant differences between wt* and wt_*pilE24*_ (Kolmogorow–Smirnow test), where the mean rupture forces with standard errors were $F_{rupture}^{wt}$ = 40.9 ± 0.9 pN and $F_{rupture}^{pilE24}$ = 38.3 ± 1.0 pN, respectively (Fig 3B). The deviation from previously published results for the wt* [27] most likely results from a different distance of the traps. Interestingly, the wt_*pilE17*_ and wt_*pilE32*_ variants exhibit much lower rupture forces of $F_{rupture}^{pilE17}=3.8\pm 0.1$ pN and $F_{rupture}^{pilE32}=6.1\pm 0.3$ pN, respectively. Moreover, the probability that a pair of bacteria shows attractive interactions is lower for all *pilE* variant strains compared to wt* (Fig ix in S1 Text).

The attractive force between bacteria initiates aggregation into microcolonies. In this study, the term “microcolony” describes spherical aggregates formed by gonococci in liquid or at the interface between the surface and liquid (Fig 3C). By the combined action of twitching motility, pilus:pilus attraction, and microcolony-fusion, wt* gonococci self-assemble within several minutes into spherical colonies comprising thousands of cells [22,24,41]. We investigated whether the different *pilE* variants support microcolony formation in this time frame. In agreement with the dual laser tweezers experiments, we found that only the strains that mediate the stronger attractive forces, wt* and wt_*pilE24*_, are capable of microcolony formation (Fig 3C). After 1 h of incubation, colonies are mostly spherical (S1 Data) with a broad distribution of colony sizes (Fig viiA in S1 Text). While many colonies are small, more than 90% of the cells reside within colonies exceeding 1,000 cells (Fig viiB in S1 Text). We note that the colony size distribution is highly dynamic because colonies are motile and can fuse [22]. By contrast, the strains wt_*pilE17*_ and wt_*pilE32*_ remain planktonic (Fig 3D and S1 Data). Since the number of pili per cell of the planktonic strains is not lower than the density of the aggregating strains (Fig iv in S1 Text), we attribute the different interaction properties to T4P stereochemistry.

**Fig 4:**
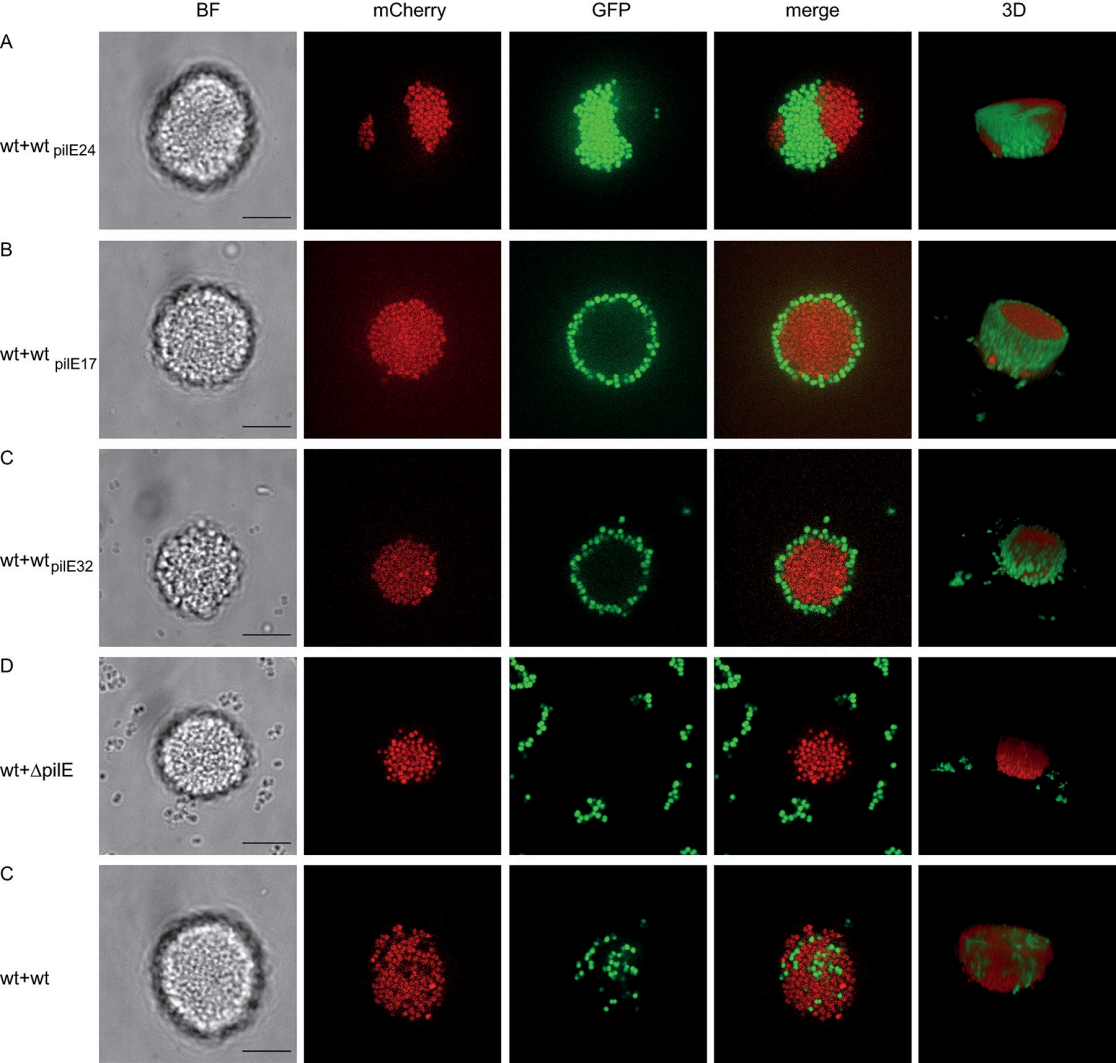
Pilin variants segregated in mixed microcolonies. wt*_red_ (Ng170) were mixed at a 1:1 ratio with (A) wt_*pilE24 green*_ (Ng309), (B) wt_*pilE17 green*_ (Ng308), (C) wt_*pilE32 green*_ (Ng310), (D) *ΔpilE*_*green*_ (Ng081), (E) wt*_green_ (Ng105). Scale bar: 10 μm. Additional biological culture replicates can be found in S1 Data.

We next addressed the question whether wt* cells and strains expressing pilin variants self-assembled into mixed microcolonies. To this end, we mixed *mcherry*-expressing wt* cells (wt*_red_) with *gfp*-expressing pilin variant strains. The 2 aggregating strains wt*_red_ and wt_*pilE24 green*_ formed colonies in which both strains segregate (Fig 4A). The most common pattern is one where each strain occupies one half of the colony. According to the differential strength of adhesion hypothesis, this phenotype is indicative of strong intrastrain interactions and weaker inter-strain interactions [28,11]. Strains wt_*pilE17 green*_ (Fig 4B) and wt_*pilE32 green*_ (Fig 4C) show comparable colony pattern when mixed with wt*_red_ cells. Both form a shell around the wt*_red_ colony made up from a single-celled layer. This morphology indicates that wt* T4P attract wt_*pilE17*_ and wt_*pilE32*_ pili, but less strongly than they attract wt*. By contrast, wt*_red_ colonies do not attract unpiliated *ΔpilE*_*green*_ bacteria (Fig 4D). These results show that while the planktonic piliated strains cannot form colonies by themselves, they can attach to existing colonies.

Taken together, these results demonstrate that different antigenic variants of the T4P have different attractive force between pairs of cells. Strongly interacting strains form colonies while weakly interacting strains remain planktonic but can associate with colonies formed by strongly interacting cells.

### The C-terminal sequence of the pilin determines the attractive force between T4P

**Fig 5:**
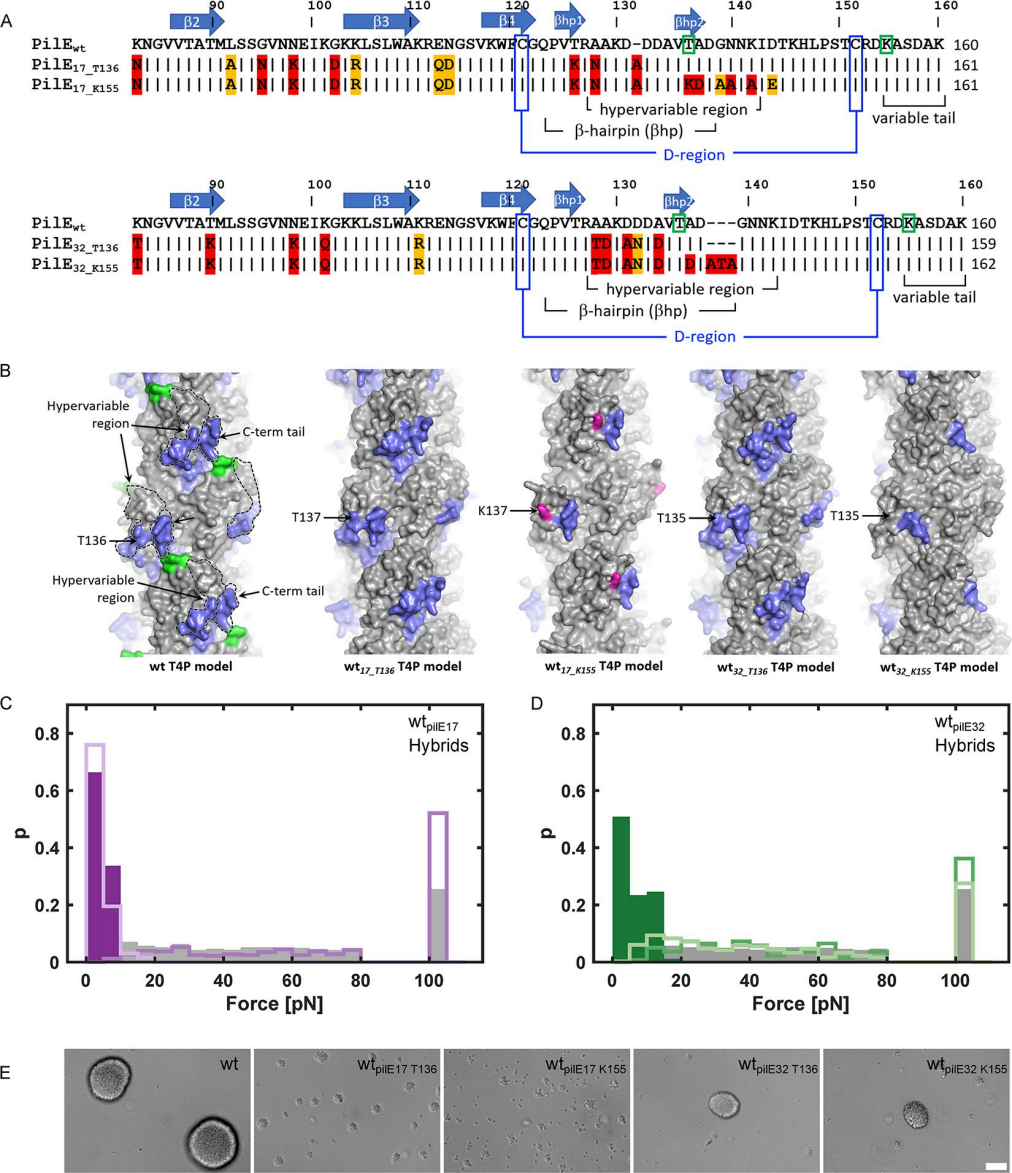
Swapping the C-terminal pilin region restores aggregation. (A) Sequences of the hybrid strains. (B) Alphafold 3 predictions of pilin monomers were used to generate filament models. The regions of PilE_wt_ that were inserted into PilE_17_ and PilE_32_, T136-160 (T136) or K155-160 (K155) are shown in blue. K137 in PilE_*17-K155*_ is colored magenta; the corresponding threonines in the other models are indicated. Probability distribution of rupture forces (C), gray: wt* (Ng150), dark purple: wt_*pilE17*_ (Ng240), purple: wt_*pilE17_T136*_ (Ng293), light purple: wt_*pilE17_T155*_ (Ng305), and (D) gray: wt* (Ng150), dark green: wt_*pilE32*_ (Ng230), green: wt_*pilE32_T136*_ (Ng295), light green: wt_*pilE32_T155*_ (Ng307). (Number of interacting cell pairs: N_wt_ = 71, N_wt*pilE17_T136*_ = 45, N_wt*pilE17_K155*_ = 54, N_wt*pilE32_T136*_ = 33, N_wt*pilE32_K155*_ = 42.) The data underlying this figure can be found in S1 Data. The linearity of the laser trap is limited to 80 pN. All rupture events exceeding this force were grouped into a single bin shown at 100 pN. (E) Typical brightfield images of gonococci in liquid culture. Scale bar: 15 μm. Additional biological culture replicates can be found in S1 Data.

We investigated which region of the pilin governs pilus:pilus interaction. The pilin structure models show high variability between the knobs and the cavities of the different pilin variants (Fig 1). We predicted that swapping the C-terminal sequence of wt_*pilE17*_ and wt_*pilE32*_, which aggregate poorly, with the corresponding sequence of wt*, which aggregates well, could restore strong interactions and aggregation to these variants. The variable knob of wt* PilE is defined approximately by residues T136 to the C-terminus, thus this segment, designated “T136”, was used to replace the corresponding C-terminal segments in the wt_*pilE17*_ and wt_*pilE32*_ PilE proteins (Fig 5A). These hybrid pilins were predicted by AlphaFold to have very similar folds to that of wt PilE (Fig viii in S1 Text). The hybrid pilins were used to generate filament models that closely resemble the model for the wt T4P (Fig 5B). The hybrid strains were strongly piliated, although the mean T4P number was slightly lower compared to the wt* strain (Fig iv in S1 Text). Rupture forces were determined for the hybrid strains wt_*pilE17_T136*_ and wt_*pilE32_T136*_ and found to be comparable to the wt* strain (Fig 5C and 5D). Furthermore, these strains aggregate and form spherical colonies (Fig 5E and S1 Data), consistent with the C-terminal segment of PilE defining the strength of pilus:pilus interactions. We note, however, that these colonies had a different size distribution compared to wt* (Fig vii in S1 Text).

Though there is considerable amino acid sequence variability in the C-terminal region of PilE, the C-terminal tail following the conserved disulfide bond is particularly disparate between the aggregating and planktonic strains. While only the last amino acid of the C-terminal tail is different between the aggregating strains wt* and wt_*pilE24*_, the tails of wt _*pilE24*_ and wt_*pilE32*_ are shorter by several residues and bear no sequence identity to each other or to PilE_wt_ or PilE_24_ (Fig 1A). To determine whether this difference affects pilus:pilus interactions, we generated the *pilE* hybrid strains wt_*pilE17_K155*_ and wt_*pilE32_K155*_, each with their C-terminal residues replaced with residues K155-K160 of PilE_wt_ (Fig 5A and 5B). This replacement did not significantly affect the number of T4P per cell (Fig iv in S1 Text). We tested their pilus rupture forces and aggregative abilities. Whereas the *pilE* hybrid strain wt_*pilE32_K155*_ showed strong pilus:pilus interactions comparable to wt*, interactions for wt_*pilE17_K155*_ are considerably weaker (Fig 5C and 5D). wt_*pilE17_K155*_ showed a stronger tendency to aggregate than wt_*pilE17*_, but the contours of the aggregates were not well defined (Fig 5E, S1 Data). wt_*pilE32_K155*_ formed spherical aggregates whose size distribution was shifted towards intermediate size colonies comprising (100 to 1,000) cells (Fig vii in S1 Text). To understand why the rupture forces of wt_*pilE17_K155*_ and wt_*pilE17*_ are comparable, but wt_*pilE17_K155*_ forms small aggregates, we addressed the question whether wt_*pilE17_K155*_ interacted more frequently in the double laser trap. We found that the fraction of randomly picked pairs of cells that exhibited pilus:pilus interaction was indeed higher for strain wt_*pilE17_K155*_ (Fig ix in S1 Text). These results show that the variable tail plays an important role in pilus:pilus interaction and aggregation, but this is not sufficient for restoring aggregation in all pilin variants.

We have previously shown that pilin posttranslational modification can affect pilus:pilus interaction [11,25]. The glycan attached to Ser63 in PilE_wt_, and likely PilE_24_, is positioned to impact the size and chemistry of the holes that govern the aggregative property of the T4P. PilE_17_ and PilE_32_ lack the Ser63. Therefore, we addressed the question whether loss of pilin glycosylation inhibits colony formation. To this end, we deleted the gene *pglF* encoding the flippase required for pilin glycosylation [42] and found that the colony phenotype was unchanged in all strains (Fig x in S1 Text). This finding shows that the loss of posttranslational modification at Ser63 is not the reason for loss of interaction of strains wt_*pilE17*_ and wt_*pilE32*_.

In summary, we find that the C-terminal region of the aggregating strain wt*, which defines the knob-hole structure, restores aggregation to both non-aggregating strains wt_*pilE17*_ and wt_*pilE32*_, whereas the hypervariable tail of wt* restores aggregation in only wt_*pilE32*_.

### Gonococci generating different pilin variants show different growth kinetics

**Fig 6:**
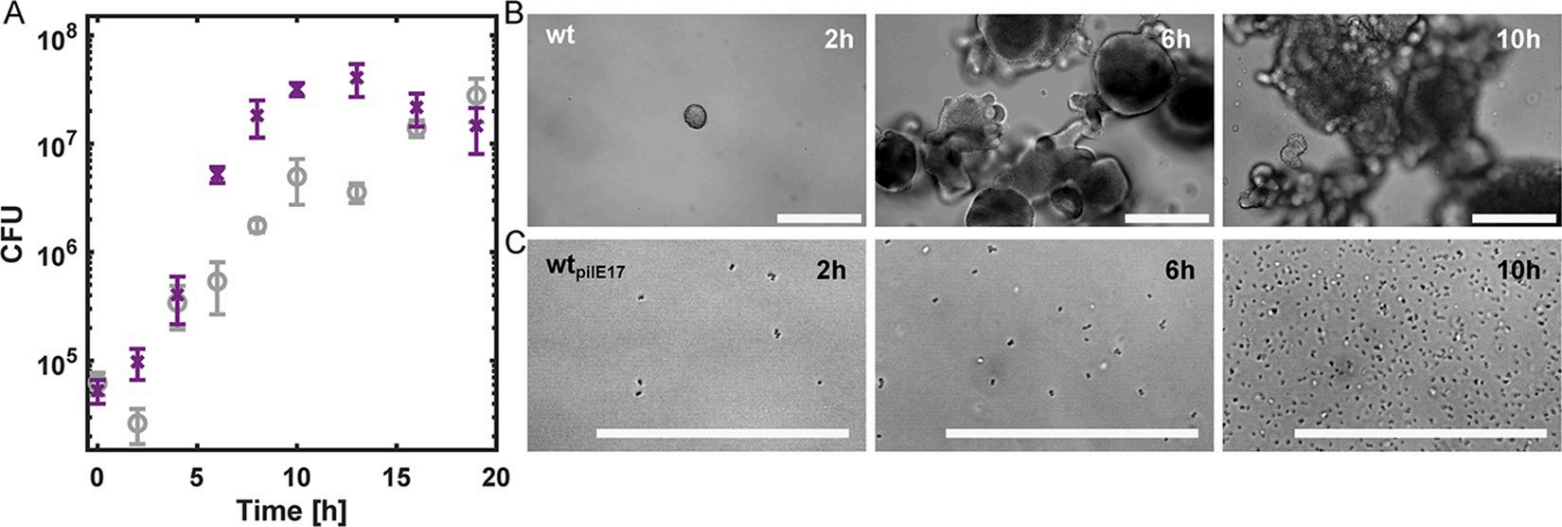
Growth kinetics of planktonic and aggregating strains. (A) CFU of the wt* strain (gray) and the wt_*pilE17*_ strain (purple). Shown are mean values and standard errors of 3 to 4 biological culture replicates. Typical brightfield images of (B) wt* and (C) wt_*pilE17*_ populations during growth in liquid culture. Samples were pipetted from the bottom of the microtiter plates and transferred to microscopy plates for imaging. Scale bar: 100 μm.

We tested whether the 2 distinct phenotypes, aggregating and planktonic, correlate with bacterial growth. First, we imaged the cells at different time points during growth. The wt* strain formed spherical colonies whose size increased with time due to growth and fusion (Fig 6B). Over time, the wt* colonies formed networks while strain wt_*pilE17*_ remained planktonic (Fig 6C). We characterized the growth kinetics by determining the colony forming units (CFUs) as a function of time for strains wt* and wt_*pilE17*_ (Fig 6A). The aggregating strain shows a detectable lag phase, whereas the planktonic strain resumes growth immediately after inoculation. After 2 h, both the planktonic and the aggregating strains exhibit exponential growth. After approximately 10 h, the planktonic strain enters into the stationary phase, while the aggregating strain continued to grow up to 19 h. The growth kinetics of wt_*pilE24*_ was comparable to wt* and the kinetics of wt_*pilE32*_ was reminiscent of wt_*pilE17*_ (Fig xi in S1 Text). To evaluate whether the exponential growth rate depends on the lifestyle, we determined the growth rates of aggregating strains (pooled for wt* and wt_*pilE24*_) and planktonic strains (pooled for wt_*pilE17*_ and wt_*pilE32*_) (Fig xi in S1 Text). The aggregating strains grow at a rate of $r^{aggre.}=0.67\pm 0.05$ h^-1^, which is significantly lower than the rate of planktonic strains $r^{plankt.}=0.92\pm 0.05$ h^-1^.

It has been reported previously that loss of T4P enhances both the growth rate of *N*. *gonorrhoeae* [15] and transcription of metabolic genes [43]. This increase can be caused by the fact that pilin generation or pilus biogenesis consumes energy, reducing the growth rate [11] or by the fact that piliated gonococci form colonies in which central bacteria are growth arrested [44]. In this study, all strains are similar in their piliation levels, yet the aggregating wt and wt_*pilE24*_ demonstrate very different growth behavior from the planktonic wt_*pilE17*_ and wt_*pilE32*_. These data indicate that aggregation strongly affects growth.

### Antigenic variants of pilin affect antibiotic tolerance but not resistance

**Fig 7:**
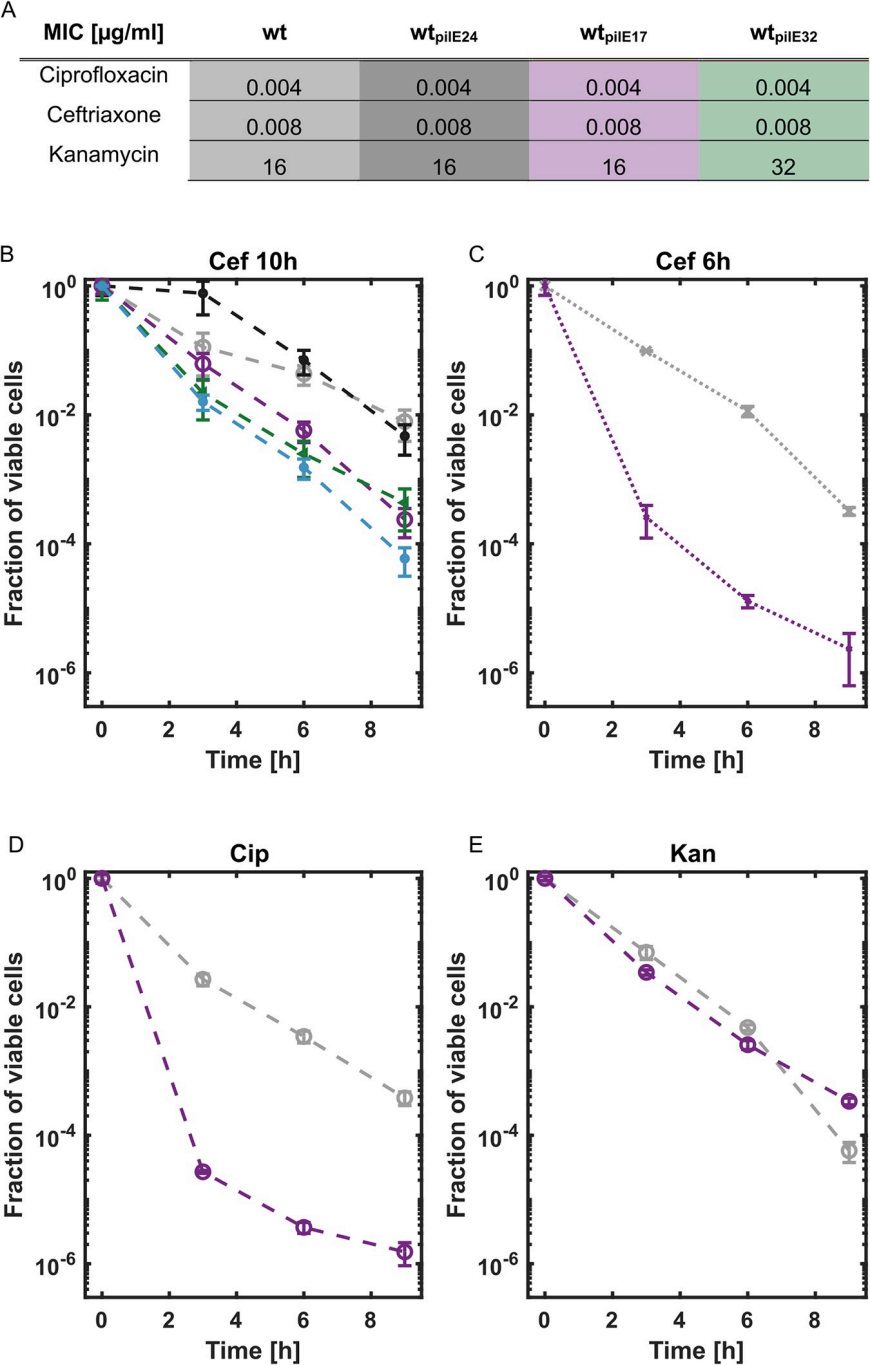
Effects of microcolony formation on antibiotic resistance and tolerance. (A) MICs for all pilin variants and antibiotic treatments were determined as the modal value from 3 biological culture replicates. (B) Fraction of viable cells (CFU normalized to CFU at the start of treatment) as a function of time during treatment with ceftriaxone (4.8 μg/ml), starting at 10 h of growth. Combined *p*-values (see Methods): p_wt-wt*pilE24*_ = 0.34, p_wt-wt*pilE17*_ = 8.8 × 10^−5^_,_ p_wt-wt*pilE32*_ = 0.00035, p_wt-ΔpilE_ = 5.3 × 10^−5^. (C) Fraction of viable cells as a function of time during treatment with ceftriaxone (4.8 μg/ml), starting at 6 h of growth. p_wt-wt*pilE17*_ = 0.00025. (D) Fraction of viable cells during treatment with ciprofloxacin (2.4 μg/ml). p_wt-wt*pilE17*_ = 8.28 × 10^−5^. (E) Fraction of viable cells during treatment with kanamycin (240 μg/ml). p_wt-wt*pilE17*_ = 0.69. Gray: wt, dark gray: wt_*pilE24*_, purple: wt_*pilE17*_, green: wt_*pilE32*_, blue: *ΔpilE*. For (B–E), mean and standard error over 3 to 5 biological culture replicates are shown. The data underlying this figure can be found in S1 Data. CFU, colony forming unit; MIC, minimal inhibitory concentration.

Next, we assessed whether variation of *pilE* affects antibiotic susceptibility, i.e., the ability to grow at elevated levels of antibiotics. We determined the minimal inhibitory concentrations (MICs) of antibiotics with different targets, in particular cell wall synthesis (ceftriaxone), DNA gyrase/topoisomerase (ciprofloxacin), and the ribosome (kanamycin) (Fig 7A). Despite the differences in aggregative behavior, there is no significant difference in MICs between the variant strains.

To characterize antibiotic tolerance, i.e., the ability to survive antibiotic concentrations higher than the MIC for extended periods of time, we examined the killing kinetics during treatment with lethal doses of antibiotics. Ceftriaxone is currently recommended for treatment of gonorrhea [45] and, therefore, we started by investigating its effects on survival during the planktonic and microcolony lifestyles. We let gonococci grow for 10 h in liquid media as described above and then added ceftriaxone at 4.8 μg/ml, which is 600× the MIC. The corresponding growth and survival curves in the absence of antibiotic treatment are shown in Fig 6. We found that the planktonic strain wt_*pilE17*_ was killed significantly faster than the aggregating strain wt* (Fig 7B). To determine whether this difference was caused uniquely by aggregation or whether the T4P per se played a role in tolerance, we characterized the killing kinetics in a *pilE* deletion strain. *ΔpilE* cells were killed slightly but significantly faster than the wt_*pilE17*_ strain, indicating that T4P or pilin have a protective role against ceftriaxone mediated killing. The killing kinetics of strain wt_*pilE24*_ was comparable to wt* and that of wt_*pilE32*_ and was comparable to wt_*pilE17*_ (Fig 7B).

To verify that the antibiotic concentration is not a limiting factor for killing, we investigated the time-kill kinetics at 3 different ceftriaxone concentrations (2.4 μg/ml, 4.8 μg/ml, and 9.6 μg/ml) and found that the aggregating strains are more tolerant than the planktonic strains independent of the ceftriaxone concentration (Fig viiA–C in S1 Text). Concluding, tolerance is not due to the aggregating cells having a lower antibiotic dose per cell, as their cell density is in fact lower after 10 h of growth than that of the planktonic cells, meaning their antibiotic dose per cell is even higher (Fig 6A). This suggests that we might underestimate the protective effect of aggregation. Since the killing kinetics were qualitatively independent of the antibiotic concentration, we conclude that the antibiotic dose per cell plays a minor role in this assay. Next, we tested whether the growth phase affects the protective effect of aggregation (Fig 7C). We treated the planktonic strain, wt_*pilE17*,_ and the aggregating strains, wt*, at 6 h of growth, i.e., in the middle of exponential growth with ceftriaxone at 600× MIC (4.8 μg/ml). We observed that the protective effect of aggregation, observed for wt*, was even stronger compared to treatment after 10 h of growth. We conclude that aggregation makes gonococci more tolerant to ceftriaxone treatment while the presence of pilin has a minor effect.

Finally, we addressed tolerance against bactericidal antibiotics with different cellular targets, in particular DNA gyrase/topoisomerase (ciprofloxacin), and the ribosome (kanamycin) at 10 h of growth (Fig 7D and 7E) [46,47]. We tested ciprofloxacin at 600× MIC (2.4 μg/ml) and kanamycin at 15× MIC (240 μg/ml) as the latter is not soluble at concentrations corresponding to 600× MIC. For ciprofloxacin (Fig 7D), the difference between the aggregating and the planktonic strain is even more pronounced than for ceftriaxone, with aggregating wt* exhibiting substantially higher viability than planktonic wt_*pilE17*_ cells. Notably, the planktonic cells showed a bi-phasic killing curve reminiscent of persister cells [32]. Under kanamycin treatment, the killing kinetics of aggregating and planktonic strains were comparable (Fig 7E). Nonetheless, the observed differences in tolerance to ceftriaxone and ciprofloxacin show that survivability of *N*. *gonorrhoeae* depends on its pilin antigenic variants, because the variation modulates T4P-mediated aggregation.

## Discussion

This study explored the effect of pilin antigenic variation on the biophysical characteristics of the Type 4 pili and its interplay with bacterial survival under antibiotic treatment. We reveal how pilin antigenic variants govern the bacterial lifestyle, as hypermutable pilin variants possess distinct aggregating or planktonic phenotypes. Explicitly, *pilE* aggregative gonococcal variants exhibit a fitness advantage when treated with bactericidal antibiotics ceftriaxone and ciprofloxacin. Our results highlight the close relationship between aggregation and tolerance and suggest that antigenic variation plays an important role in bacterial survival and persistence that extends beyond escaping immune surveillance.

By investigating the pilin sequences of clinical isolates, we found *pilE* variants with distinct pilus functionalities. For example, wt_*pilE17*_ shows more efficient motility and aggregates less efficiently than wt*. We demonstrate here that these differences are due to T4P stereochemistry and not to piliation levels, in contrast to previous findings [7]. How might stereochemistry support different T4P-mediated functions? We propose that the undulating surface of the pilus allows intimate interactions along its length via protruding knobs formed by the β-hairpins and C-terminal tails inserting into the holes between subunits in adjacent pili. For strong pilus:pilus interactions, these features would need to have stereochemical complementarity. From the structural models, it is clear that amino acid changes in these knobs or holes can profoundly affect their shape and chemistry and thus their complementarity. Thus, an amino acid change on one feature may require a compensatory change on the other for stereochemical complementarity to be maintained. PilE_wt_ and PilE_24_ are most similar in sequence. PilE_24_ differs from PilE_wt_ for a number of surface residues, both at the rim of the hole and on the knob, but since strain wt_*pilE24*_ is a microcolony former like wt*, these differences are likely compensatory. The knobs of PilE_wt_ and PilE_24_ are somewhat negatively charged, which would complement their positively charged holes, but this could also be said of PilE_17_ and PilE_32_. Since the latter are not aggregating, it may be that while their knobs and holes are electrostatically complementary their shapes/structures are not. We show here that the C-terminal region, from the β-hairpin through C-terminal tail, defines pilus:pilus interactions, as insertion of this segment, T136, in the non-aggregating strains restores aggregation. A shorter segment corresponding to the C-terminal tail only, restores the high rupture force and aggregation for wt_*pilE32*_ but is less effective for wt_*pilE17*_. Interestingly, PilE in the wt_*pilE17-K155*_ has an exposed lysine, K137, on the β-hairpin, whereas wt* and wt_*pilE17-T136*_, wt_*pilE32-T136*_, and wt_*pilE32-K155*_ all have a threonine at this position (Fig 5B). Kennouche and colleagues found that a charged patch centered on an exposed lysine (K140), which is located in the loop immediately following the β-hairpin, is crucial for aggregation of *N*. *meningitidis* [17]. By contrast, in our strain wt_*pilE17-K155*_ a single exposed lysine on the β-hairpin is linked to poor aggregation. This comparison shows that it is difficult to ascribe pilus:pilus interaction to single amino acid residues, since pilin antigenic variation generates too much sequence diversity. Importantly, however, both studies show that the highly variable C-terminal region of the pilin is crucial for aggregation.

In this study, we replaced the *pilE* sequence with sequences from clinical isolates. While the *pilS* that determine these structures are similar between the strains (Fig i in S1 Text), we cannot exclude that epistatic effects select for integration of different *pilS* sequences in different strains. For example, multiple genes encoding for T4P related proteins and other surface structures are phase variable [48]. Depending on the presence (or absence) of T4P assembly components and other surface structures, different *pilE* sequences may be selected for during infection. Our study does not account for such epistatic effects, since the *pilE* sequences were exchanged between different strains with different phase varions. In future studies, it will be interesting to address such epistatic effects.

It is widely accepted that biofilm formation leads to higher tolerance against antibiotic treatment [49–51], but the mechanisms causing tolerance are poorly understood. *N*. *gonorrhoeae* form microcolonies in solution that have properties similar to bacterial biofilms, including a gradient of limited oxygen, of growth, and of tolerance against antibiotics [44,52], and thus allow a systematic characterization of the effects of bacterial aggregation on tolerance. Here, we established stable *N*. *gonorrhoeae* strains expressing non-aggregative T4P that are otherwise fully functional, thus serving as excellent planktonic control strains. We show that T4P-mediated aggregation has a strong effect on tolerance. For ceftriaxone, we found an order of magnitude increase in the fraction of viable cells when bacteria formed colonies. This effect was robust with respect to the antibiotic concentration and the growth phase. Ceftriaxone is a β-lactam and for this class of antibiotics it has been shown for *E*. *coli* that the killing rate is inversely correlated with growth rate [53]. Similarly, growth-rate dependent killing rates were reported for *E*. *coli* treated with ciprofloxacin [49]. Bacteria at the center of gonococcal microcolony are growth arrested [44], and the fraction of dead cells in this location is lower under ceftriaxone treatment compared to the edges of the colonies [27], suggesting that local growth arrest enhances tolerance. Moreover, we propose that the formation of oxygen gradients within gonococcal colonies [52] can protect bacteria residing at the microcolony center where the oxygen concentration is lower. There is evidence that bactericidal antibiotics including ciprofloxacin and ceftriaxone kill bacteria (at least partially) by producing reactive oxygen species [50,51]. Reduced antibiotic penetration is suggested to enhance tolerance [36]. We can exclude this mechanism for our system, since we showed previously that antibiotic treatment causes swelling of the cell body and this effect is homogenous throughout the colonies [27]. Unexpectedly, microcolony formation did not protect gonococci from kanamycin treatment. For early-stage colonies, we found that cells at the center of the colonies are more tolerant to kanamycin than peripheral cells, most likely because cells at the center have lower electrical membrane potential [52]. On the hand, central cells grow more slowly and it has been shown that antibiotics that irreversibly bind the ribosome (like kanamycin) are more effective for slow-growing bacteria [54]. It is unclear, however, whether a similar argument holds for the killing rate at kanamycin concentrations exceeding the MIC. We anticipate that the strains introduced in this study will be useful for studying tolerance against stresses other than antibiotics. For example, it has been shown that piliation protects gonococci from killing by hydrogen peroxide [55,56], antimicrobial peptides [56], and neutrophils [55]. The fully piliated strains with self-aggregating versus non-aggregating properties will allow us to distinguish between protection by self-aggregation and protection by other T4P-related functions.

Based on our results, we propose that pilin antigenic variation has various functions beyond its well-known role in escape from immune surveillance. Within a gonococcal population, antigenic variation rapidly generates a standing variation of different *pilE* sequences with phenotypes that support adhesion, aggregation, twitching motility, or DNA uptake. We have shown that different variants can form colonies comprising different variants that segregate in agreement with the differential strength of adhesion hypothesis [28]. This shows that pilin antigenic variation governs the structural organization within the colonies and we anticipate that it impacts the efficiency of horizontal gene transfer across different variants. While the generation of variations is likely random, variants with different phenotypes are selected for during infection by the host environment. Here, we showed that these phenotypic changes impact antibiotic tolerance and, therefore, antigenic variation plays a key role in microcolony diversification, infection, and treatment of gonorrhea.

## Materials and methods

### Growth conditions

We used the same growth conditions described in previous studies [27]. Gonococcal base agar was made from 10 g/l dehydrated agar (BD Biosciences, Bedford, MA), 5 g/l NaCl (Roth, Darmstadt, Germany), 4 g/l K_2_HPO_4_ (Roth), 1 g/l KH_2_PO_4_ (Roth), 15 g/l Proteose Peptone No. 3 (BD Biosciences), 0.5 g/l soluble starch (Sigma-Aldrich, St. Louis, MO), and supplemented with 1% IsoVitaleX (IVX): 1 g/l D-glucose (Roth), 0.1 g/l L-glutamine (Roth), 0.289 g/l L-cysteine-HCL x H_2_O (Roth), 1 mg/l thiamine pyrophosphate (Sigma-Aldrich), 0.2 mg/l Fe(NO_3_)_3_ (Sigma-Aldrich), 0.03 mg/l thiamine HCl (Roth), 0.13 mg/l 4-aminobenzoic acid (Sigma-Aldrich), 2.5 mg/l β-nicotinamide adenine dinucleotide (Roth), and 0.1 mg/l vitamin B12 (Sigma-Aldrich). GC medium is identical to the base agar composition but lacks agar and starch.

### Bacterial strains

All strains used in this study (Table i in S1 Text) were derived from the *opa-* selected VD300 strain [57] and carry a deletion of the G4 motif required for pilin antigenic variation [58]. Colonies were grown overnight on agar plates and each colony used for the experiments was inspected using a stereomicroscope to ensure that it maintained its *opa-* phenotype.

Clinical isolates NG17, NG24 were cultured from urethral swabs in 2016 and NG32 in 2017, from male patients presenting with urethritis. After identification and susceptibility testing, they were stored in glycerol at −80°C. Isolates were recovered from the freezer by plating out on chocolate agar for the purpose of this study.

First, clinical isolates were transferred back into the piliated state as described in the following. We grew bacterial cells for 1 day in liquid medium (37°C and 5% CO_2_) without shaking. The next day, we transferred bacteria growing at the surface with a loop to fresh GC-media. This process was repeated until a pellicle at the surface was formed. Then, we plated the pellicle on GC agar plates. Piliated phenotypes were chosen according to the colony morphology and testing for twitching motility.

To determine the sequence of the *pilE* variants, we performed a PCR with primers sk5 and sk34 on the respective gDNA (isolated with the Blood and Tissue Kit, Qiagen). The PCR product was sequenced with primer pilE_IWupstream_ (Eurofins).

### Construction of *pilE* variant strains

In order to construct isogenic strains that differ solely in the *pilE* sequence, the *pilE* sequences of the clinical isolates were cloned into the ΔG4 background strain (Ng150, derivative of MS11, [59]) replacing the native *pilE* gene. To avoid further genetic modification, the *pilE* genes were introduced by *ermC-rpsL*_*s*_ based clean insertion as described [18].

The process for the construction was identical for strains wt_pilE17_, wt_pilE24_, and wt_pilE32_ except for the respective primers. First, the 5′ UTR region of *pilE* was amplified from gDNA of strain Ng150 using primers sk159 and sk160 (Table iii in S1 Text). Second, the *pilE* gene was amplified from gDNA of the different clinical isolates NG17, NG24, and NG32 with primers sk161 and sk175, sk161 and sk167, and sk161 and sk162, respectively. Third, the 3′ UTR of *pilE* including the *ermC-rpsL*_*s*_ was amplified from gDNA of strain Ng225 using primers sk163 and sk158. The 3 PCR products were fused and the final product was spot transformed into strain Ng150. After selection on erythromycin, insertions were controlled via screening PCR with primers sk32 and sk45. The respective strains were named *ΔG4 pilE*NG17/24/32 clean insertion step 1 (Ng239, Ng241, Ng229, respectively).

Next, the fusion construct for the counter selection was generated. To this end, the 5′ UTR region including the newly introduced *pilE* genes was amplified from gDNA of strains *ΔG4 pilE*NG17/32/24 clean insertion step 1 (Ng239, Ng229, Ng241) with primers sk159 and sk176 (Table iii in S1 Text), sk159 and sk168, sk158 and sk165, respectively. The 3′ UTR of *pilE* was amplified from gDNA of strain Ng150 with primers sk164 and sk158. The 2 products were joined in a fusion PCR and transformed into the respective strain of the first step (Ng239, Ng241, Ng229). During selection on streptomycin, the *ermC*-*rpsL*_s_ construct is spliced out and the mutants are isogenic to the parental strain (Ng150) except for the *pilE* sequences. Insertions were controlled via screening PCR with primers sk32 and sk45 and subsequently checked via sequencing with primer sk129 (Eurofins). The final strains are referred to as wt_*pilE24*_ (Ng242), wt_*pilE17*_ (Ng240), and wt_*pilE32*_ (Ng230).

### Construction of *pilE* hybrid strains

*pilE*_*T136*_ hybrids: The N-terminal part of the *pilE* gene of strain wt_*pilE17*_ (Ng240) and wt_*pilE32*_ (Ng230) was fused with the C-terminal part of *pilE* of the wt* (Ng150) starting at amino acid T136. In detail, amino acids K137-E160 of *pilE*_*17*_ and T135-P158 of *pilE*_*32*_ were replaced by T136-K160 of *pilE*_*wt*_. To this end, the N-terminal part unil K137 of *pilE*_*17*_ and T136 *pilE*_*32*_ including 567 bp upstream were amplified from wt_*pilE17*_ (Ng240) or wt_*pilE32*_ (Ng230) using primers sk384 and sk385 or sk384 and sk388, respectively (Table iii in S1 Text). The T136 C-terminal part including the *ermC rpsL*_*s*_ cassette and the downstream part of *pilE* were amplified from strain *T126C step1* (Ng225, [18]) using primers sk386 and sk390 (*pilE*_*17*_) or sk389 and sk390 (*pilE*_*32*_). Both PCR products were fused with the GXL-polymerase (TaKaRa). The final fusion product was transformed into either wt_*pilE17*_ (Ng240) or wt_*pilE32*_ (Ng230). Selection was achieved by plating transformants on agar plates containing erythromycin. Correct insertion was checked via screening PCR (sk5 and sk32) and sequencing (sk5 or sk32). The mutants were named wt_pilE17_T136_ step1 (Ng292) and wt_pilE32_T136_ step1 (Ng294).

For the second step of the clean replacement, the fused *pilE* gene (*pilE*_*17_T136*_ or *pilE*_*32_T136*_) was amplified from the respective first step, either wt_pilE17_T136_ step1 (Ng292) or wt_pilE32_T136_ step1(Ng294) using primers sk384 and sk131. The downstream region of *pilE* was amplified with primers sk132 and sk390 from wt* (Ng150). The PCR products were fused with the GXL-polymerase and the fusion product was transformed in either wt_pilE17_T136_ step1 (Ng292) or wt_pilE32_T136_ step1(Ng294). Afterwards, the strains were selected on streptomycin. Correct insertion was checked via screening PCR and sequencing with primers sk5 and sk32. The new strains were named wt_pilE17_T136_ (Ng293) or wt_pilE32_T136_ (Ng295).

*pilE*_*K155*_ hybrids: The C-terminal tail of the *pilE* gene of strains wt_*pilE17*_ (Ng240) and wt_*pilE32*_ (Ng230) were replaced with the C-terminal tail starting at K155 of *pilE* of strain wt* (Ng150). The *ermC-rpsL*_*s*_ cassette was amplified from strain *T126C* step1 [18] and fused with the corresponding *pilE* upstream and downstream regions. The fusion product was transformed into either wt_*pilE17*_ (Ng240) or wt_*pilE32*_ (Ng230) and selected on erythromycin.

The *pilE*_*17/32*_ gene including 567 bp upstream were amplified from wt_*pilE17*_ (Ng240) or wt_*pilE32*_ (Ng230) using primers sk384 and sk391 or sk384 and sk393, respectively (Table iii in S1 Text). The K155 C-terminal tail including the *ermC rpsL*_*s*_ cassette and the downstream part of *pilE* were amplified from strain *T126C* step1 (Ng225) using primers sk392 and sk390 (NG17) or sk394 and sk390 (NG32). Both PCR products were fused with the GXL-polymerase (TaKaRa). The final fusion product was transformed into wt_*pilE17*_ (Ng240) or wt_*pilE32*_ (Ng230) and after transformation selection was performed on plates containing erythromycin. Correct insertion was checked via screening PCR with primers sk5 and sk32 and sequencing (sk5 or sk32) resulting in strains wt_pilE17_K155_ step1 (Ng304) and wt_pilE32_K155_ step1(Ng306).

For the second step of the clean replacement, the hybrid *pilE*s were amplified from the respective first step wt_pilE17_K155_ (Ng304) or wt_pilE32_K155_ step1 (Ng306). Primers sk384 and sk131 (wt_pilE17_K155_) and primer sk384 and sk395 (wt_pilE32_K155_) were used. The downstream region of *pilE* was amplified with primers sk132 and sk390 from wt* (Ng150). The PCR products were fused with the GXL-polymerase (TaKaRa) and the fusion product was transformed in either wt_pilE17_K155_ step1 (Ng304) or wt_pilE32_K155_ step1 (Ng306). Then, the strains were selected on streptomycin. Correct insertion was checked via screening PCR and sequencing (sk5 or sk32) and the strains were named wt_pilE17_K155_ (Ng305) and wt_pilE32_K155_ (Ng307).

### Construction of *ΔpglF* strains

Deletion of the flippase *pglF* strongly reduces pilin glycosylation by interrupting the membrane translocation of lipid-attached carbohydrates [42]. To delete *pglF*, the respective strains were transformed with genomic DNA of strain Ng156 and selected on kanamycin [11].

### Construction of *gfp* expressing strains

Genomic DNA of strain Ng105 [11] was used for transformation to insert GFP into the chromosome of the respective strains, generating strains wt_*pilE24 green*_ (Ng309), wt_*pilE17 green*_ (Ng308), and wt_*pilE32 green*_ (Ng310) (Table i in S1 Text). Transformants were selected on plates containing erythromycin.

### Construction of *ΔpilE strain*

The *pilE* gene was interrupted with a kanamycin resistance cassette. Three individual DNA fragments were amplified via PCR and fused; 5′ *pilE* including the upstream *pilE* region was amplified with primers sk45 and sk46 from gDNA of strain Ng150. *kanR* was amplified with primers sk47 and sk48 from genomic DNA of strain Ng052 [60]; 3′ *pilE* and the downstream region of *pilE* was amplified using primers sk49 and sk50. The PCR products were fused and the fusion construct was transformed into wt* strain. Transformants were selected on kanamycin.

### Identification of *pilS* copies in genomes of gonococcal clinical isolates

To verify that the *pilE* variants are products of pilin antigenic variation, we first determined the *pilS* copies in the genome of the clinical isolates. *pilS* were identified using annotated *pilS* copies of strain *N*. *gonorrhoeae* MS11 (NCBI, CP003909.1) as reference. End and start sites of *pilS* copies were determined either by matching blast results using the SnapGene software (www.snapgene.com) or blastn (BLAST, NCBI) of the respective MS11 orthologs. The blast results were verified manually by identifying the conserved cysteine regions *cys1* and *cys2* in each *pilS* copy. The sequence identity of *pilS* copies from clinical isolates to *pilS* copies from strain *N*. *gonorrhoeae* MS11 were obtained *via* blastn (BLAST, NCBI).

### Twitching motility analysis

We let the strains grow for 12 to 16 h on GC agar plates. We picked a few colonies, resuspended them in liquid GC medium, transferred them to a BSA coated coverslip (1 mg/ml), and then sealed the sample with Valep (Vaseline, wool fat and paraplast in a ratio of 1:1:1). Subsequently, we recorded videos with a confocal Ti-E inverted microscope (Nikon) equipped with a thermobox at 37°C with a framerate of 10 Hz and 100× magnification over 30 s. We stopped each measurement after 15 min to ensure constant experimental conditions. All strains were characterized in biological culture replicates on at least 3 different days.

Next, we tracked each bacterium over the whole measurement time. From each track, we calculated the MSD from the displacements $\delta (\tau )=|\vec{r}(t+\tau )-\vec{r}(t)|$ [61]. As the bacteria follow a corrleated random walk, we fitted $<\delta^{2}(\tau )>=2\tau_{c}v^{2}(\tau -\tau_{c}(1-e^{\left(-\tau /\tau_{C}\right)}$) + A for the first 5 s to determine the correlation time τ_c_ and velocity *v* to the data of a single track (Fig vi in S1 Text). The variable A accounts for the tracking error. We took the mean and standard variation of the fit parameters to determine the average correlation time τ and velocity *v*_*corr*_ for the *pilE* variants. Statistical analysis of the distributions of the fit parameters for single bacteria tracks was performed via the Mann–Whitney U test.

### Transmission electron microscopy and determination of T4P number

Bacteria grown overnight on GC agar plates were resuspended in liquid medium and adjusted to an optical density (OD_600_) of 0.1. For sample preparation, 10 μl of the bacterial solution was transferred on a 100 mesh formvar coated copper grid (Science Services) and incubated for 20 min at room temperature. For fixation, the grid was put upside down in a drop of 2% formaldehyde (Science Services) and incubated for 5 min, followed by 5 times washing in PBS. Next, the mesh was placed on a drop of 1% glutaraldehyde (Sigma) for 5 min and washed 8 times in Milli-Q water. The samples were blotted on filter paper. For negative staining, the cells were again incubated with 10 μl of uranyl acetate for 4 min and the spill-over was removed by filter paper. Then, they were imaged in a transmission electron microscope (JEM-2100Plus (JEOL)) in the imaging facility of the CECAD, Cologne. All images were taken at 6,000× magnification at 40 μm under the default focus at room temperature. Pili were counted manually. Only pili that could be assigned to a single cell were considered. If single pili were not distinguishable within T4P bundles, they were counted as one pilus. All strains were characterized in biological culture replicates on 1 to 3 different days.

### Generation of PilE and pilus models

AlphaFold [39] was used to generate models of PilE_wt_ and the PilE varients. Models were superimposed upon Chain A of the *N*. *gonorrhoeae* T4P cryoEM reconstruction [40] in Chimera (Petterson and colleagues, PMID 15264254) and residues 1–48 of the model were replaced with that of Chain A to replace the continuous α1-helix of the AF models with the melted helix seen in the filament. The helical symmetry parameters of *N*. *gonorrhoeae* T4P (10.1 Å rise, 100.8° rotation) were imposed on the PilE models to generate 18-mer filament models. Electrostatic potential was generated using the APBS tool of PyMol [62].

### Confocal microscopy

Bacteria grown overnight on GC agar plates were resuspended in GC liquid media to an optical density of OD_600_ 0.1. Then, cells were mixed in a 1:1 ratio and vortexed vigorously. Subsequently, the bacterial solution was incubated for 45 min in a shaking incubator (37°C, 250 rpm, 5% CO_2_) to let the cells aggregate. Next, 300 μl of the suspension was transferred into a Poly-L-lysine coated (Sigma, final concentration: 0.005%) Ibidi treat 8-well plate. All images were acquired using an inverted microscope (Ti-E, Nikon) equipped with a thermo box (37°C) and a spinning disk unit (CSU-1, Yokogawa) with 100× magnification, 1.49 NA, oil immersion objective lens. The excitation wavelengths were 488 nm and 560 nm. Three-dimensional z-stacks with a plane-to-plane distance of 0.2 μm and an overall height of 10 to 12 μm were acquired for 3D images. All mixtures were characterized in biological culture replicates on at least 3 different days.

### Acquisition of brightfield images

Brightfield images of cells were recorded with an inverted Nikon Eclipse Ti with an ORCA camera model (40× magnification). After different time periods, 300 μl of growing cell cultures were transferred into an Ibidi 8-well plate attached to cover glass and were imaged directly.

To verify microcolony formation in liquid culture, cells grown overnight on GC agar plates were adjusted to an optical density of 0.1 in GC media, and 100 μl of this culture was transferred into 200 μl GC media in an Ibidi 8-well plate attached to a cover glass. Then, the cells were incubated for up to 1.5 h (37°C, 5% CO_2_) before imaging.

### Distribution of aggregate sizes

The distribution of aggregate sizes was determined by incubating the respective strains at an initial OD_600_ of 0.033 in an Ibidi 8-well plate attached to a cover glass for 1 h and imaging using brightfield imaging (Fig vii in S1 Text). Aggregates were segmented using the Fiji segmentation tool and the radius was determined from the area of the segments assuming that aggregates were spherical. Strains wt_*pilE17*_, wt_*pilE32*_ did not form aggregates. Strain wt_*pilE17K155*_ had a stronger tendency to aggregate than wt_*pilE17*_, but the aggregates did not show clearly defined colony shapes, and therefore, we did not attempt to determine the radii of these aggregates (Fig 5E). Therefore, these 3 strains were excluded from the analysis. All strains were characterized in biological culture replicates on at least 3 different days.

We estimated the number of cells per aggregate $N=\Phi V_{agg}/V_{bacterium}$ assuming that the aggregates are spherical and that the bacteria are spheres with a radius of 0.5 μm. The volume fraction of gonococci is Φ≈0.5 [63].

### Dual laser tweezers experiments

The interaction forces of *pilE* variants were determined via a dual laser trap. The experimental setup and analysis is already published [22]. In short, we resuspended a few bacterial colonies from overnight GC agar plates in liquid GC medium. We added 1:1,000 ascorbic acid (500 mM). Next, we inoculated the bacteria on a BSA coated cover slip (1 mg/ml) and sealed the slide with VALEP (Vaseline, wool fat and paraplast in a ratio of 1:1:1). The major building blocks of the laser tweezers setup consist of a microscope equipped with a thermo-box at 33°C, an IR-laser (1,064 nm) and an acousto-optical deflector which creates 2 time-shared optical potentials. The trap distance was set to 2.64 μm. We acquired videos of interacting bacteria with a framerate of 50 Hz. After 15 min, we stopped the measurement as we observed decreased activity of the gonococci. All strains were characterized in different samples on at least 3 different days.

We detected the displacements *d* of the bacteria from the equilibrium positions via a Hough transformation algorithm. From the displacement tracks, we determined the forces (*F ~ d*) and identified the interaction states, as described earlier in [22]. The potential of each trap was approximated to be harmonic for forces up to 80 pN.

At 100% laser intensity, the traps showed a trap stiffness of *k*_100%_ = 0.1 pN/nm, whereby the laser intensity *I* is proportional to the stiffness of the trap *k* ~ *I*. We note that it was not possible to conduct the experiments with the same laser intensities for all variants, since deflections of the wt_*pilE32*_ and wt_*pilE17*_ strains were infrequent. This indicated that these 2 strains have lower interaction forces as they could not overcome the trapping potential of the traps at 100% laser power. Therefore, we adjusted the intensities of the laser. The measurements for the wt* and wt_*pilE24*_ could be performed at 100% laser power while for the other variants, the laser intensity needed to be decreased to 10% for strain wt_*pilE32*_ and to 5% for strain wt_*pilE17*_. Under these conditions, the probability of pilus:pilus binding (Fig ix in S1 Text) was high enough for characterizing T4P mediated attractive forces.

### Bacterial growth curves

Bacterial growth and aggregation were monitored by measuring the OD600 with an Infinite M200 plate reader. After 12 to 14 h on GC-agar plates, bacteria were resuspended in liquid GC medium and adjusted to an optical density OD_600_ of 0.1. For each time point and each condition, a 48-well plate (Greiner), containing 1 ml liquid GC media, was inoculated with 10 μl of the bacterial suspension. We incubated the bacteria at 37°C, 5% CO_2_ with a shaking period of 2 min per OD cycle, and measured the OD every 10 min. All strains were characterized in biological culture replicates on at least 3 different days.

To determine the number of CFUs during 19 h of growth, we performed the same protocol as described above and additionally transferred a whole well to a 1.5 ml reaction tube every 1 to 3 h. Next, we harvested the bacteria by centrifugation (5,000 g, 3 min) and resuspended them in 500 μl GC media. Subsequently, we vortexed for 30 s and added 500 μl of MQ-water to initiate the disassembly of gonococcal aggregates. Then, we again vortexed the suspension for 2 min. The prolonged time of vortexing was already shown to be sufficient to shear pili [64]. Additionally, we ensured reproducibility for each strain and for strains showing the same lifestyle. We performed 1:10 dilution series with vortexing inbetween and plated 50 μl of different dilutions on non-selective GC agarplates. After 48 h of growth (37°C with 5% CO_2_), we counted the CFUs.

To determine the growth rates, we plotted the growth curves from 2 h to 8 h. Then, we performed a linear regression *fitlm* for the log-plotted data via *Matlab* [61]. The slopes are defined as the growth rates. The significance analysis was performed via a pairwise ANOVA test for the linear regression models which includes an interaction term for the different *pilE* variant strains [61].

### MIC determination

The minimal inhibitory concentration of the different antibiotics (ceftriaxone, ciprofloxacin, and kanamycin) was determined for each strain, and 1 ml cultures supplemented with increasing antibiotic concentrations were inoculated with approximately 5∙10^5^ cells of the following strains, wt* (Ng150), wt_*pilE24*_ (Ng242), wt_*pilE17*_ (Ng240), and wt_*pilE32*_ (Ng230). Bacteria were grown in an Infinite M200 plate reader at 37°C, 5% CO_2_ with a shaking period of 2 min per OD cycle. The lowest concentration of an antibiotic without detectable growth (OD600 nm ≤ 0.1) after 24 h was determined as the MIC of the respective antibiotic. All strains were characterized in biological culture replicates on at least 3 different days.

### Bacterial survival assay

To investigate how antigenic variation impacts bacterial survival under antibiotic treatments, we developed a survival assay. Isolates were initially grown as described for the bacterial growth curves. Following resuspension in GC media, we let the bacteria grow for 10 h in an Infinite M200 plate reader at 37°C, 5% CO_2_ with a shaking period of 2 min per OD cycle. If other pre-growth durations were used, we indicated this in the figure and description for the specific experiments. OD was measured every 10 min. Next, we added antibiotics to each well except the control wells, and the plate was further incubated. The final concentrations of ceftriaxone were 2.4/4.8/9.6 μg/ml corresponding to the 300× MIC/600× MIC/1,200× MIC, respectively. For ciprofloxacin treatment, we added antibiotics to a final concentration of 2.4 μg/ml, and for kanamycin, the concentration was 240 μg/ml, corresponding to 600× MIC and 15× MIC, respectively. The solubility of kanamycin was too low to increase the antibiotic concentration. To determine the number of viable bacteria, cells were plated at 0 h, 3 h, 6 h, and 9 h after antibiotic treatment as described before (Methods: Bacterial growth curves). All strains were characterized in biological culture replicates on at least 3 different days.

Statistical analysis of the killing kinetics was performed via a combined *p*-values method for discrete data [65]. We have used the Mann–Whitney U test to compare single time points followed by the combination of the *p*-values via Mudholkar and George combining method [66].

## Supporting information

S1 Text Supplementary Figures and Supplementary Tables. Fig i. Sequence identity of *pilS* copies from gonococcal clinical isolates to orthologs from *N*. *gonorrhoeae* MS11. The sequence identity of each *pilS* to the respective copy of strain MS11 was determined using blastn (BLAST, NCBI). gray: *pilS*_*24*_, orange: *pilS*_*17*_, red: *pilS*_*32*_. **Fig ii. Mapping of different *pilS* to *pilE* variants.** Each *pilS* copy of the clinical isolate was aligned against the respective *pilE* variant (black: *pilE*_wt*_, dark gray: *pilE*_*24*,_ red: *pilE*_*32*,_ orange: *pilE*_*17*_). *pilS* sequences with 100% identity and at least 6 bp in length are shown. The alignments are ordered by the length of the matching sequence, e.g., the longest matching alignment for each position within *pilE* is directly underneath the *pilE* sequence. Only the 3 best matches are shown for each *pilE* variant. The illustrations were created with SnapGene software (www.snapgene.com). pilS5_extended: extended *pilS5* sequence including the conserved *cys2* region. **Fig iii. Model of the charge densities**. The charge density was simulated via PyMol and the APBS tool [62,69]. Models of wt* and variant pilus filaments are shown from the top and side in surface representation with electrostatic surface potential. Blue: positive charge, red: negative charge. **Fig iv. Pilus number per cell for *pilE* variants**. The pili numbers were determined from TEM images. Box plots show the median (central mark), bottom and top patches show 25th and 75th percentiles, respectively. Outliers are plotted individually (red + symbol) and are defined as values which are larger than 1.5 times the interquartile range from the bottom or top of the box, which corresponds to 99.3 percent coverage if the data is normally distributed, according to the Matlab function *boxplot* [61] which was used here. The whiskers length is defined as the maximum and minimum excluduing the outliers. *P*-values were determined via a rank-sum test: *p* > 0.05 for wt*, wt_*pilE24*_, wt_*pilE17*_, wt_*pilE17*_*K155*_, and wt_*pilE32_K155*_, *p* < 0.05 for wt_*pilE32*_, wt_*pilE17_T136*_, and wt_*pilE32_T136*_. Number of analyzed bacteria: N_wt*_ = 37, N_wt*pilE24*_ = 33, N_wt*pilE17*_ = 16, N_wt*pilE32*_ *=* 25, N_wt*pilE17*_*K155*_ = 18, and N_wt*pilE32_K155*_ = 18, Nwt_*pilE17_T136*_ *=* 27, and N_wt*pilE32_T136*_ *=* 19. The data underlying this figure can be found in S1 Data. **Fig v. Correlation time and velocity of twitching motility of *pilE* variant strains**. (A) Correlation time of motile cells on a BSA coated coverslide. (B) Velocity of twitching motility. Significance analysis via Mann–Whitney U test compared to the wt*, star: *p* < 0.05. Error bars: 95% confidence bounds from fit to correlated random walk model (Fig vi in S1 Data). The data underlying this figure can be found in S1 Data. **Fig vi. Mean squared displacement (MSD) for all tracks of single cells on a BSA-coated cover slide**. Strains (A) wt* (Ng150), (B) wt_*pilE24*_ (Ng242), (C) wt_*pilE17*_ (Ng240), (D) wt_*pilE32*_ (Ng230). The MSD was fitted for the time interval of the first 5 s. Gray: trajectories of individual cells, red line: MSD model with fit parameters averaged from single MSD fits to single tracks of bacteria. *N* = 46–200 trajectories per strain. The data underlying this figure can be found in S1 Data. **Fig vii. Distributions of aggregate size after 1 h of incubation with initial OD_600_ of 0.033**. (A) Cumulative probability distribution *p* of aggregate radius *r_agg_* with *r_agg_*≥2*μm*. (B) Estimated fraction of cells that reside within colonies comprising light gray: *N*<100 cells, gray: 10<*N*<1,000 cells, black: *N*>1,000 cells. Shown are only the the strains that form aggregates with well-defined contours. The data underlying this figure can be found in S1 Data. **Fig viii. Sequence alignment and structure predictions for PilE hybrids**. (A) Sequence alignment of the C-terminal regions. (B) Pilin models were generated using AlphaFold. PilE_17_T136_ (green) and PilE_32_T136_ (orange) superimposed on PilE_wt_. PilE_17_K155_ (green) and PilE_32_K155_ (orange) superimposed on PilE_wt_. **Fig ix. Fraction of successful attempts in dual trap assay**. We counted the fraction of interacting bacteria pairs because not every pair of bacteria showed interaction. This fraction strongly depends on the trap stiffness which was set to (A) *k* ≈ 0.1 pN/nm for wt*, wt_*pilE24*_, wt_*pilE17_T136*_, wt_*pilE32_T136*_, and wt_*pilE32_K155*_. Since interactions were nearly undetectable at *k* = 0.1 pN/nm for strains wt_*pilE32*_, wt_*pilE17*_ and wt_*pilE17_K155*_, the stiffnesses were reduced to *k* = 0.005 pN/nm (light red) and k ≈ 0.01 pN/nm (10%), respectively. Number of trapped bacteria pairs: *N* = (73–170). Error bars: standard error over different days. The data underlying this figure can be found in S1 Data. **Fig x. Pilin glycosylation does not impact the colony phenotype of PilE variants**. Representative images of PilE variants in a Δ*pglF* background. (A) wt* Δ*pglF* (Ng156), (B) wt_*pilE24*_ Δ*pglF* (Ng312), (C) wt_*pilE17*_ Δ*pglF* (Ng311), (D) wt_*pilE32*_ Δ*pglF* (Ng313). Scale bar: 50 μm. Fig xi. Growth rates for strains wt* (Ng150), wt_*pilE24*_ (Ng242), wt_*pilE17*_ (Ng240), wt_*pilE32*_ (Ng230). (A) Growth curves for all *pilE* variants from counting colony forming units. *N* = 3–4. Error bars: standard errors. (B, D) Linear regression fits to logarithmic data of CFU counts of each strain or pooled data regarding the lifestyle: planktonic or aggregating. (C, D) Growth rates determined from fits in (B) and (D), respectively. Error bars: errors of the fits. ANOVA test of the linear regression model indicated no significant difference between the growth rates of the individual strains with p_wt-wt*pilE24*_ = 0.995, p_wt-wt*pilE17*_ = 0.071, p_wt-wt*pilE32*_ = 0.074 but significant differences for pooled data p_aggreg.-planktonic_ = 0.0074. The data underlying this figure can be found in S1 Data. **Fig xii. Survival assay with different concentrations of ceftriaxone after 10 h of growth**. Killing kinetics of all variants, gray: wt*, dark gray: wt_*pilE24*_, purple: wt_*pilE17*_, and green: wt_*pilE32*_, for (A) 300× MIC, combined *p*-values (see Methods): p_wt-wt*pilE24*_ = 0.63, p_wt-wt*pilE17*_ = 0.00029, p_wt-wt*pilE32*_ = 0.0011; (B) 600× MIC, p_wt-wt*pilE24*_ = 0.35, p_wt-wt*pilE17*_ = 8.8×10^−5^, p_wt-wt*pilE32*_ = 0.00035; (C) 1,200× MIC. p_wt-wt*pilE24*_ = 0.99, p_wt-wt*pilE17*_ = 0.0038, p_wt-wt*pilE32*_ = 0.0013. Shown are mean and standard error over 3 to 4 biological culture replicates. The data underlying this figure can be found in S1 Data. **Table i. Strains used in this study. Table ii. Amino acid sequence identities of complete and partial regions of *pilE* compared to the MS11 *pilE* amino acid sequence according to Fig 1. The amino acid sequences were compared using BLAST [70]. Table iii. Primers used in this study**.
(PDF)

S1 Data Data used for generating the figures. The data sheet contains the data used for generating Figs 3B, 5C,D, 6A, 7, and Figs iv, v, vi, vii, ix, xi, and xii in S1 Text and additional micrographs for Figs 3C, 4, and 5E.
(XLSX)

## Abbreviations

CFU: colony forming unit
MIC: minimal inhibitory concentration
T4P: Type 4 pili
TEM: transmission electron microscopy
