# Supplementary material for: Pilin antigenic variants impact gonococcal lifestyle and antibiotic tolerance by modulating interbacterial forces
Source: PLoS Biol. 2025 Jan 30;23(1):e3003022. doi: 10.1371/journal.pbio.3003022 (PMC11813099; doi:10.1371/journal.pbio.3003022)
Supplement: S1 Text — The sequence identity of each pilS to the respective copy of strain MS11 was determined using blastn (BLAST, NCBI). gray: pilS24, orange: pilS17, red: pilS32. Fig ii. Mapping of different pilS to pilE variants. Each pilS copy of the clinical isolate was aligned against the respective pilE variant (black: pilEwt*, dark gray: pilE24, red: pilE32, orange: pilE17). pilS sequences with 100% identity and at least 6 bp in length are shown. The alignments are ordered by the length of the matching sequence, e.g., the longest matching alignment for each position within pilE is directly underneath the pilE sequence. Only the 3 best matches are shown for each pilE variant. The illustrations were created with SnapGene software (www.snapgene.com). pilS5_extended: extended pilS5 sequence including the conserved cys2 region. Fig iii. Model of the charge densities. The charge density was simulated via PyMol and the APBS tool [62,69]. Models of wt* and variant pilus filaments are shown from the top and side in surface representation with electrostatic surface potential. Blue: positive charge, red: negative charge. Fig iv. Pilus number per cell for pilE variants. The pili numbers were determined from TEM images. Box plots show the median (central mark), bottom and top patches show 25th and 75th percentiles, respectively. Outliers are plotted individually (red + symbol) and are defined as values which are larger than 1.5 times the interquartile range from the bottom or top of the box, which corresponds to 99.3 percent coverage if the data is normally distributed, according to the Matlab function boxplot [61] which was used here. The whiskers length is defined as the maximum and minimum excluduing the outliers. P-values were determined via a rank-sum test: p > 0.05 for wt*, wtpilE24, wtpilE17, wtpilE17_K155, and wtpilE32_K155, p < 0.05 for wtpilE32, wtpilE17_T136, and wtpilE32_T136. Number of analyzed bacteria: Nwt* = 37, NwtpilE24 = 33, NwtpilE17 = 16, NwtpilE32 = 25, NwtpilE17_K155 = [file pbio.3003022.s001.pdf]

Supporting Information for

Pilin antigenic variants impact gonococcal lifestyle and antibiotic tolerance by modulating interbacterial forces

Isabelle Wielert[1,2,§], Sebastian Kraus-Römer[1,2,§], Thorsten E. Volkmann[1,2], Lisa Craig[3], Paul G. Higgins[2,4,5], Berenike Maier[1,2,*]

[1]Institute for Biological Physics, University of Cologne, Germany

[2]Center for Molecular Medicine Cologne

[3]Department of Molecular Biology and Biochemistry, Simon Fraser University, Burnaby, British Columbia, Canada

[4]Institute for Medical Microbiology, Immunology and Hygiene, Faculty of Medicine and University Hospital Cologne, University of Cologne, Cologne, Germany

[5]German Centre for Infection Research, Partner site Bonn-Cologne, Cologne, Germany

[§] equal contribution

***** corresponding

Supporting Figures

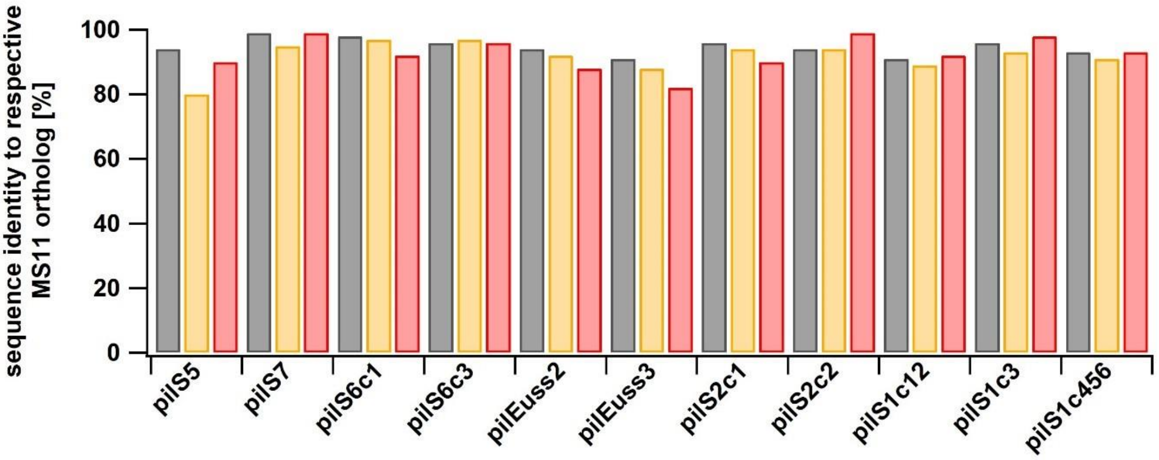

Figure i. Sequence identity of *pilS* copies from gonococcal clinical isolates to orthologs from *N. gonorrhoeae* MS11. The sequence identity of each *pilS* to the respective copy of strain MS11 was determined using blastn (BLAST®, NCBI). grey: $pilS_{24}$, orange: $pilS_{17}$, red: $pilS_{32}$.

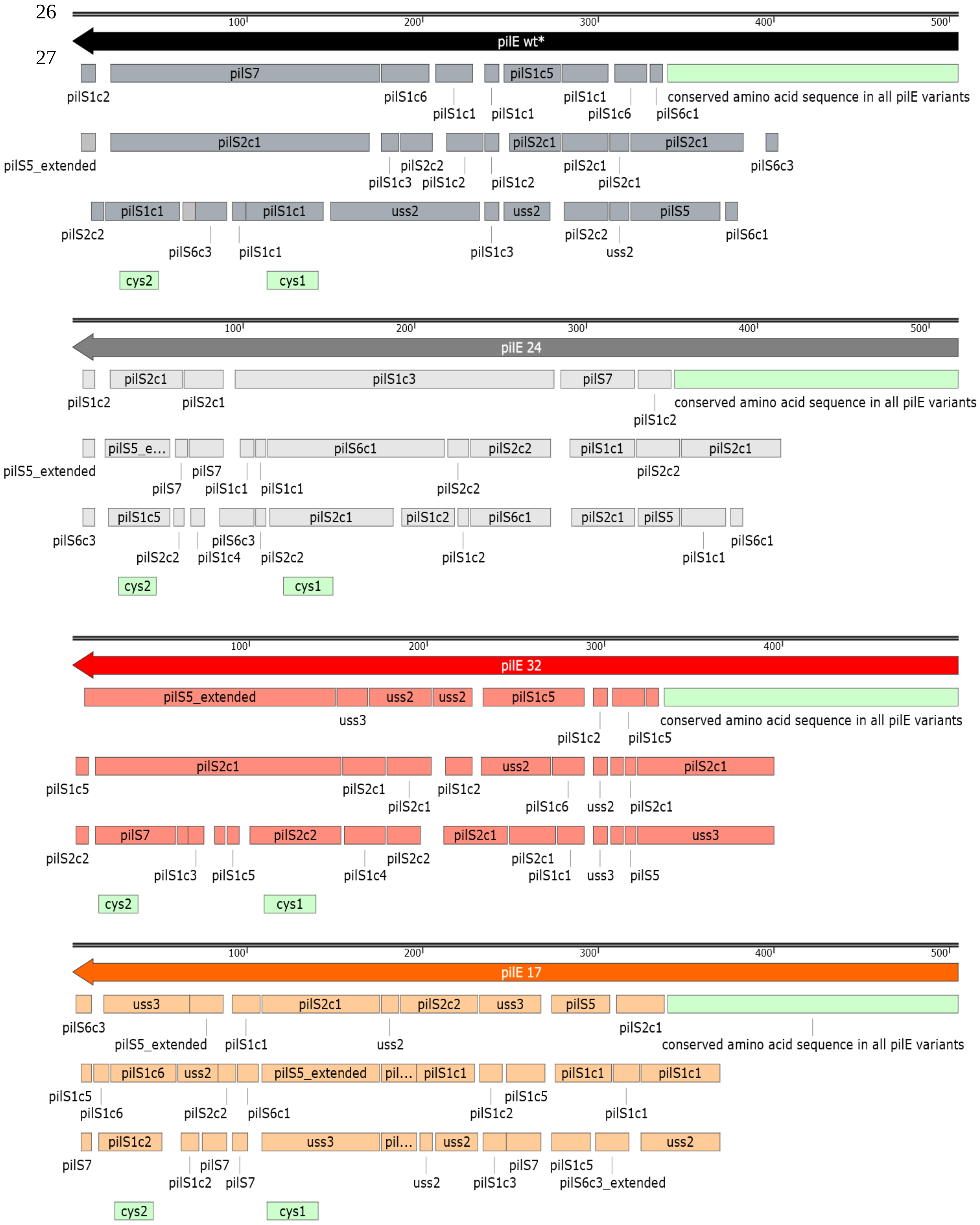

100
200
300
400
500
pilE wt*
pilS7
pilS1c5
pilS1c2
pilS1c6
pilS1c1
pilS1c1
pilS1c1
pilS1c6
conserved amino acid sequence in all pilE variants
pilS6c1
pilS2c1
pilS2c1
pilS2c1
pilS5_extended
pilS2c2
pilS1c3
pilS1c2
pilS1c2
pilS2c1
pilS2c1
pilS6c3
pilS1c1
pilS1c1
uss2
uss2
pilS5
pilS2c2
pilS6c3
pilS1c1
pilS1c3
pilS2c2
uss2
pilS6c1
cys2
cys1
100
200
300
400
500
pilE 24
pilS2c1
pilS1c3
pilS7
pilS1c2
pilS2c1
conserved amino acid sequence in all pilE variants
pilS1c2
pilS5_e…
pilS6c1
pilS2c2
pilS1c1
pilS2c1
pilS5_extended
pilS7
pilS2c2
pilS7
pilS1c1
pilS1c1
pilS2c2
pilS1c5
pilS2c1
pilS1c2
pilS6c1
pilS2c1
pilS5
pilS6c3
pilS6c3
pilS6c1
pilS2c2
pilS1c4
pilS2c2
pilS1c2
pilS1c1
cys2
cys1
100
200
300
400
pilE 32
pilS5_extended
uss2
uss2
pilS1c5
uss3
conserved amino acid sequence in all pilE variants
pilS1c2
pilS1c5
pilS2c1
uss2
pilS2c1
pilS1c5
pilS2c1
pilS1c2
pilS2c1
pilS1c6
uss2
pilS2c1
pilS7
pilS2c2
pilS2c1
uss3
pilS2c2
pilS2c2
pilS2c1
pilS1c3
pilS1c5
pilS1c4
pilS1c1
uss3
pilS5
cys2
cys1
100
200
300
400
500
pilE 17
uss3
pilS2c1
pilS2c2
uss3
pilS5
pilS6c3
pilS2c1
pilS5_extended
pilS1c1
uss2
conserved amino acid sequence in all pilE variants
pilS1c6
uss2
pilS5_extended
pil…
pilS1c1
pilS1c1
pilS1c1
pilS1c5
pilS1c5
pilS1c6
pilS2c2
pilS6c1
pilS1c2
pilS1c1
pilS1c2
uss3
pil…
uss2
uss2
pilS7
pilS7
pilS7
pilS1c5
pilS1c2
pilS7
uss2
pilS1c3
pilS6c3_extended
cys2
cys1

Figure ii. Mapping of different *pilS* to *pilE* variants. Each *pilS* copy of the clinical isolate was aligned against the respective *pilE* variant (black: *pilE*$_{wt*}$, dark grey: *pilE*$_{24,}$ red: *pilE*$_{32,}$ orange: *pilE*$_{17}$). *pilS* sequences with 100 % identity and at least 6 bp in length are shown. The alignments are ordered by the length of the matching sequence, e.g. the longest matching alignment for each position within *pilE* is directly underneath the *pilE* sequence. Only the three best matches are shown for each *pilE* variant. The illustrations were created with SnapGene software (www.snapgene.com). pilS5_extended: extended *pilS5* sequence including the conserved *cys2* region.

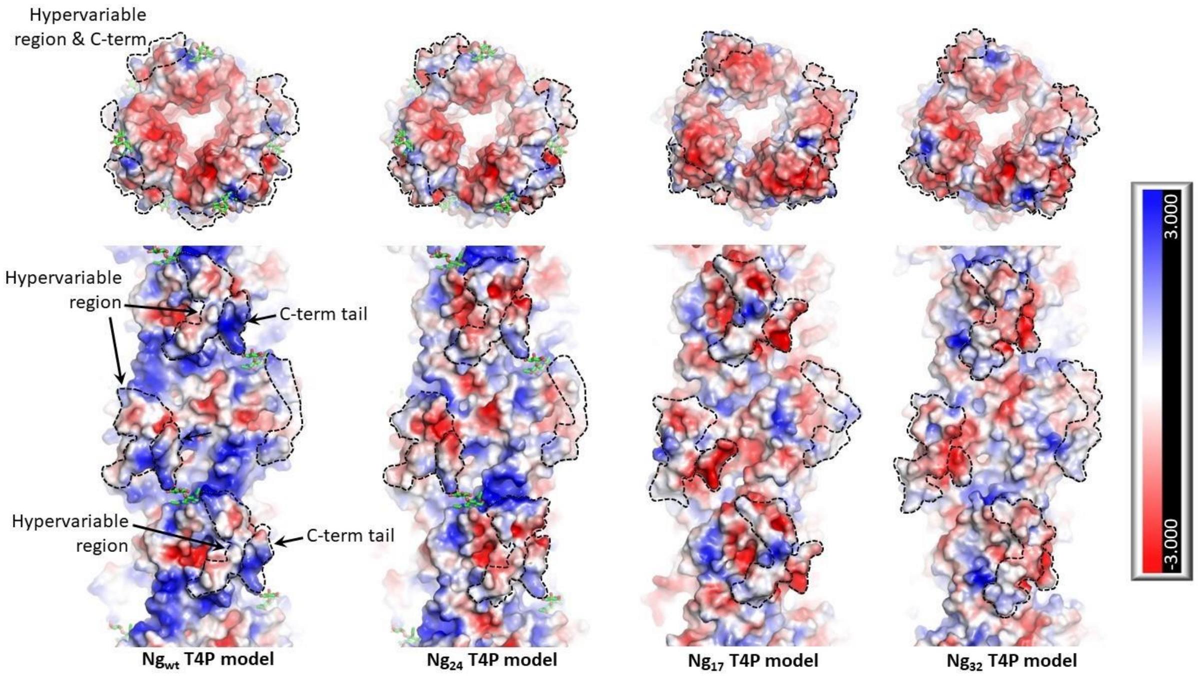

Figure iii. Model of the charge densities. The charge density was simulated via PyMol and the APBS tool [65,69]. Models of wt* and variant pilus filaments are shown from the top and side in surface representation with electrostatic surface potential. Blue: positive charge, red: negative charge.

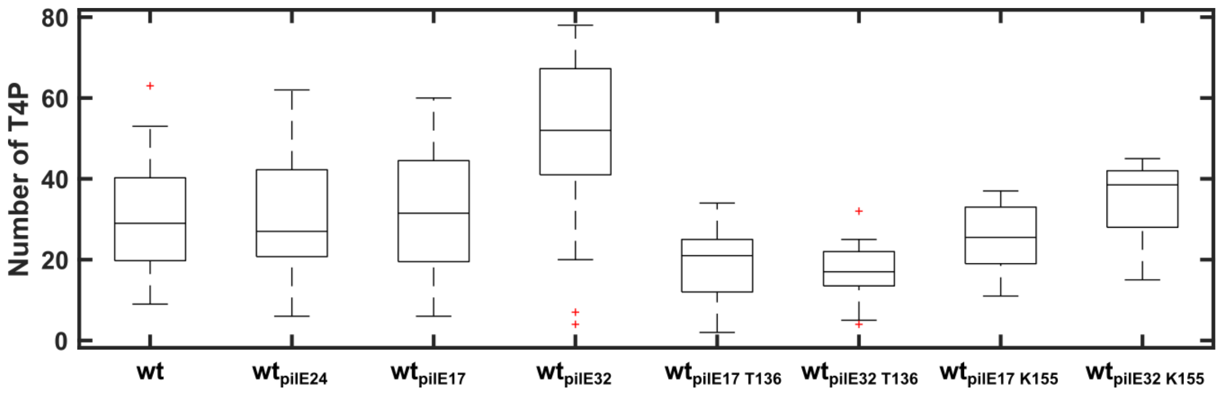

Figure iv. Pilus number per cell for *pilE* variants. The pili numbers were determined from TEM images. Box plots show the median (central mark), bottom and top patches show 25th and 75th percentiles, respectively. Outliers are plotted individually (red + symbol) and are defined as values which are larger than 1.5 times the interquartile range from the bottom or top of the box, which corresponds to 99.3 percent coverage if the data is normally distributed, according to the Matlab function *boxplot* [64] which was used here. The whiskers length is defined as the maximum and minimum excluduing the outliers. P-values were determined via a rank-sum test: $p > 0.05$ for wt*, wt_{pilE24}, wt_{pilE17}, wt_{pilE17_K155}, and wt_{pilE32_K155}, $p < 0.05$ for wt_{pilE32}, wt_{pilE17_T136}, and wt_{pilE32_T136}. Number of analysed bacteria: $N_{wt*} = 37$,$N_{wtpilE24} = 33$,$N_{wtpilE17} = 16$, $N_{wtpilE32} = 25$, $N_{wtpilE17\_K155} = 18$, and $N_{wtpilE32\_K155} = 18$, $Nwt_{pilE17\_T136} = 27$, and $N_{wtpilE32\_T136} = 19$. The data underlying this figure can be found in S1 Data.

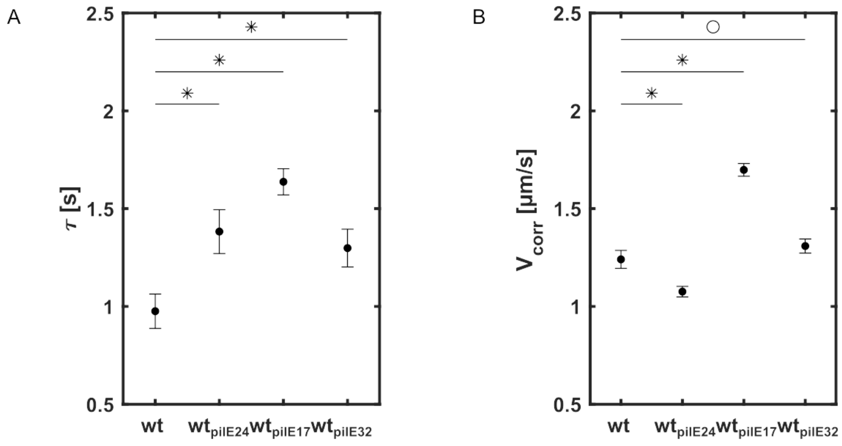

Figure v. Correlation time and velocity of twitching motility of *pilE* variant strains. A) Correlation time of motile cells on a BSA coated coverslide. B) Velocity of twitching motility. Significance analysis via Mann-Whitney U test compared to the wt*, star: $p < 0.05$. Error bars: 95% confidence bounds from fit to corrrelated random walk model (Fig. S6). Error bars: 95% confidence bounds from fit to correlated random walk model (Fig. vi in S1 Data). The data underlying this figure can be found in S1 Data.

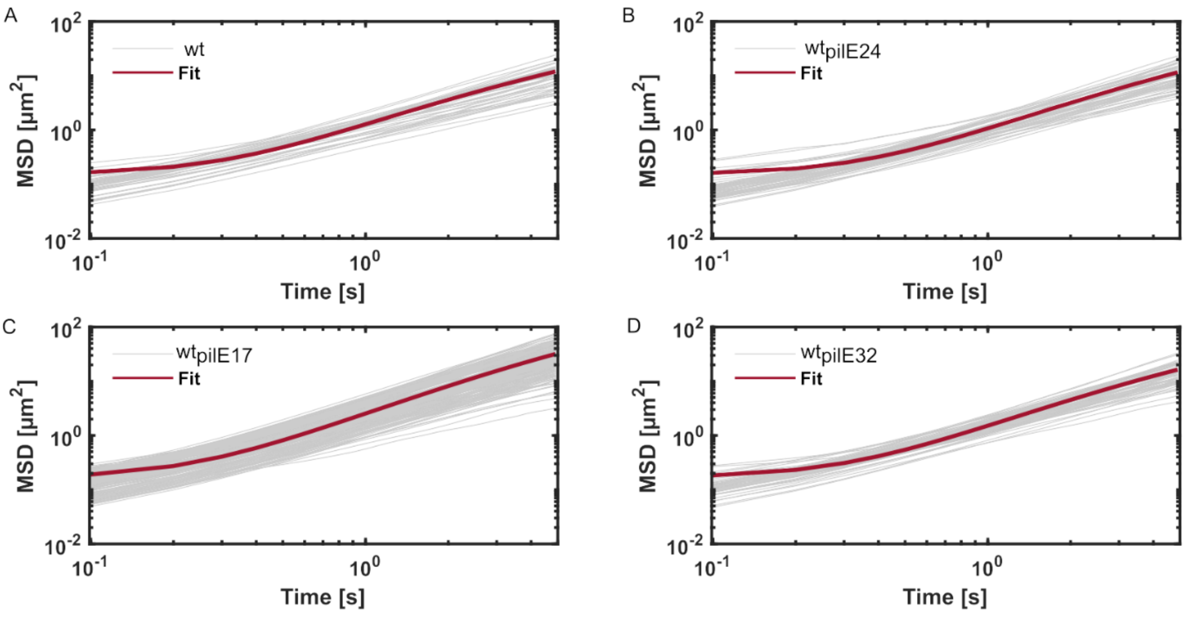

Figure vi. Mean squared displacement (MSD) for all tracks of single cells on a BSA-coated cover slide. Strains A) wt* (Ng150), B) wt$_{\textit{pilE24}}$ (Ng242), C) wt$_{\textit{pilE17}}$ (Ng240), D) wt$_{\textit{pilE32}}$ (Ng230). The MSD was fitted for the time interval of the first 5 s. Grey: trajectories of individual cells, red line: MSD model with fit parameters averaged from single MSD fits to single tracks of bacteria. N = 46-200 trajectories per strain. The data underlying this figure can be found in S1 Data.

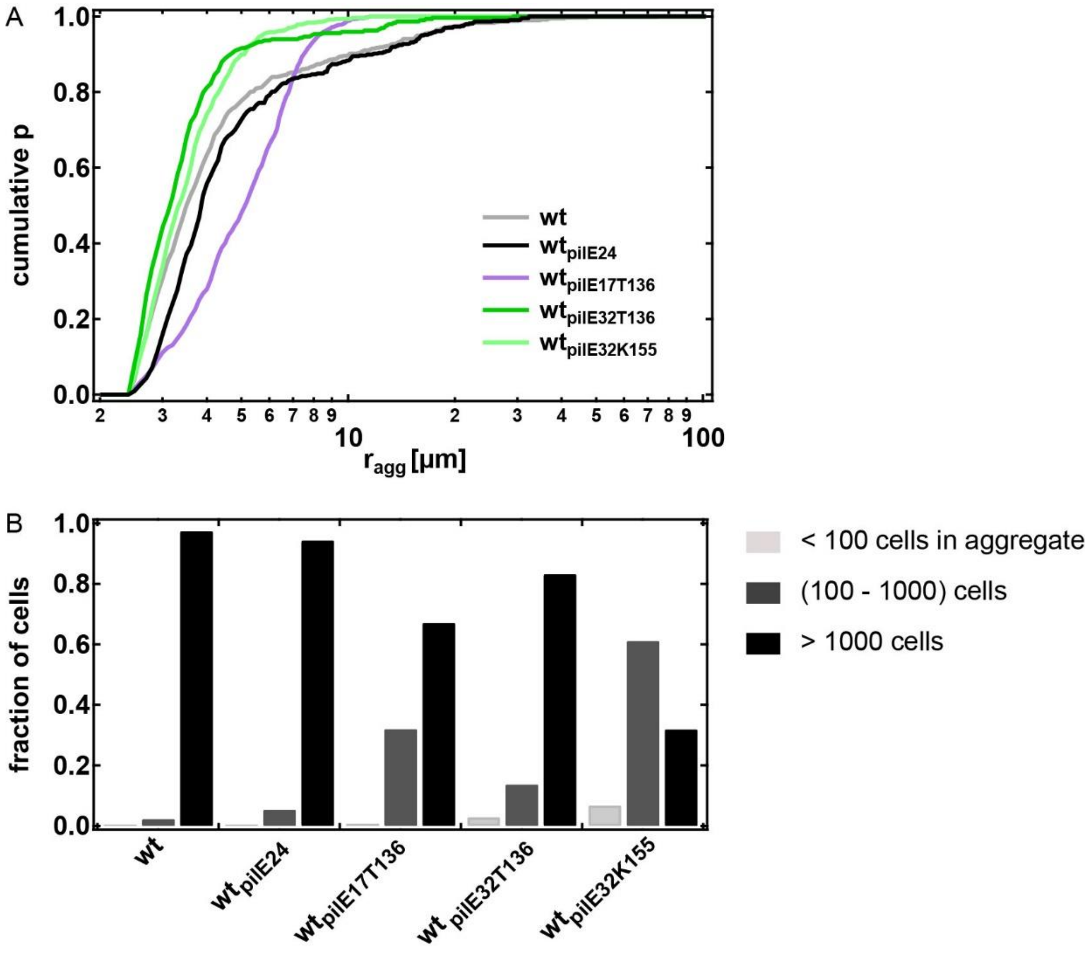

Figure vii. Distributions of aggregate size after 1 h of incubation with initial OD_{600} of 0.033. A) Cumulative probability distribution p of aggregate radius r_{agg} with $r_{agg} \geq 2\mu m$. B) Estimated fraction of cells that reside within colonies comprising light gray: $N < 100$ cells, gray: $10 < N < 1000$ cells, black: $N > 1000$ cells. Shown are only the the strains that form aggregates with well defined contours. The data underlying this figure can be found in S1 Data.

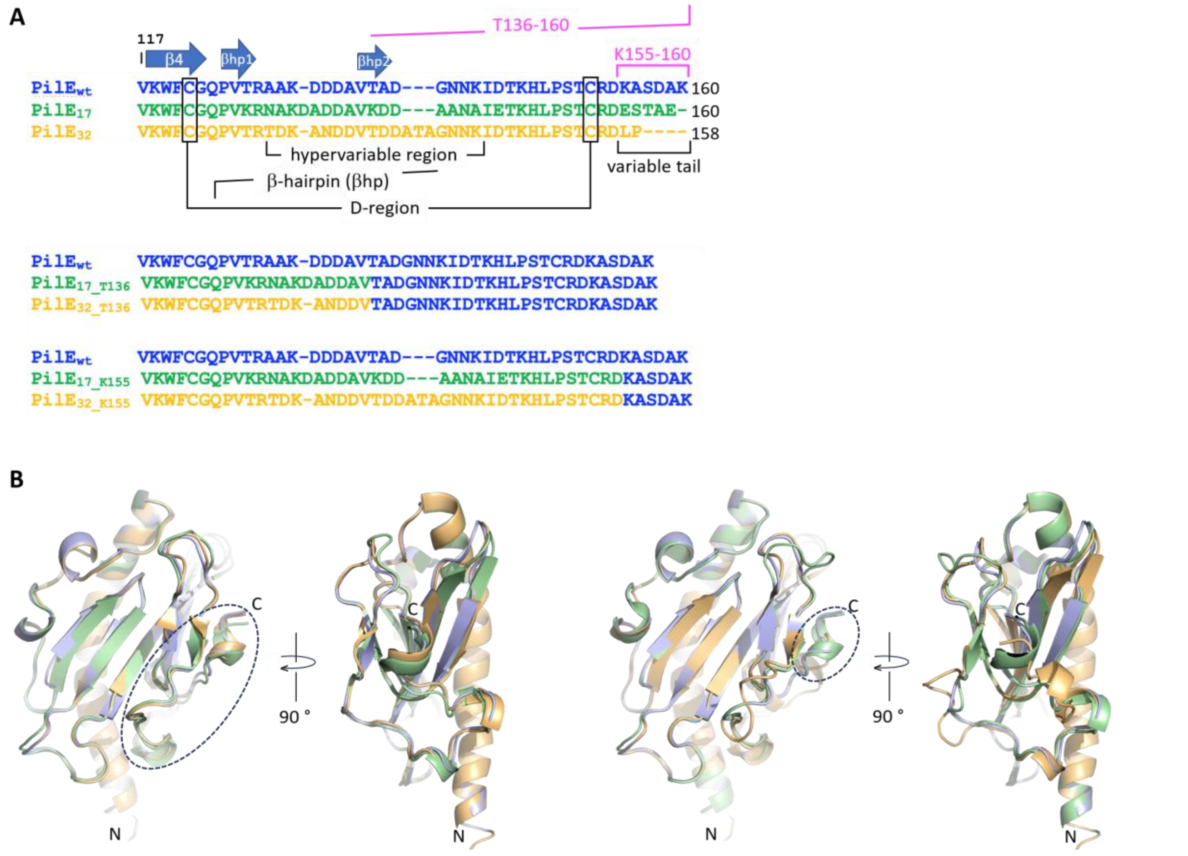

$PilE_{17\_T136}$ (green) and $PilE_{32\_T136}$ (orange) superimposed on $PilE_{wt}$ $PilE_{17\_K155}$ (green) and $PilE_{32\_K155}$ (orange) superimposed on $PilE_{wt}$

Figure viii. Sequence alignment and structure predictions for PilE hybrids. A) Sequence alignment of the C-terminal regions. B) Pilin models were generated using AlphaFold. $PilE_{17\_T136}$ (green) and $PilE_{32\_T136}$ (orange) superimposed on $PilE_{wt}$. $PilE_{17\_K155}$ (green) and $PilE_{32\_K155}$ (orange) superimposed on $PilE_{wt}$.

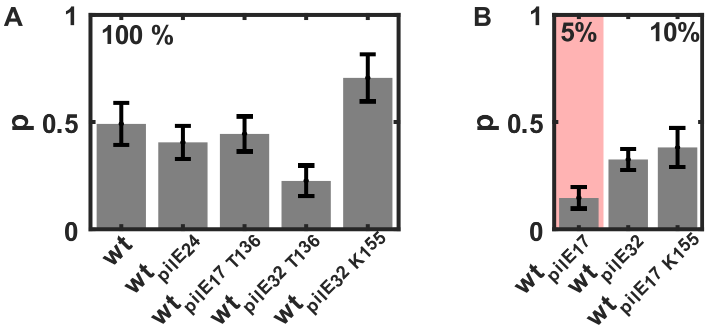

Figure ix. Fraction of successful attempts in dual trap assay. We counted the fraction of interacting bacteria pairs because not every pair of bacteria showed interaction. This fraction strongly depends on the trap stiffness which was set to A) $k \approx 0.1$ pN/nm for wt*, wt$_{pilE24}$, wt$_{pilE17_T136}$, wt$_{pilE32_T136}$, and wt$_{pilE32_K155}$. Since interactions were nearly undetectable at $k = 0.1$ pN/nm for strains wt$_{pilE32}$, wt$_{pilE17}$ and wt$_{pilE17_K155}$, the stiffnesses were reduced to $k = 0.005$ pN/nm (light red) and k ≈ 0.01 pN/nm (10%), respectively. Number of trapped bacteria pairs: N = (73 – 170). Error bars: standard error over different days. The data underlying this figure can be found in S1 Data.

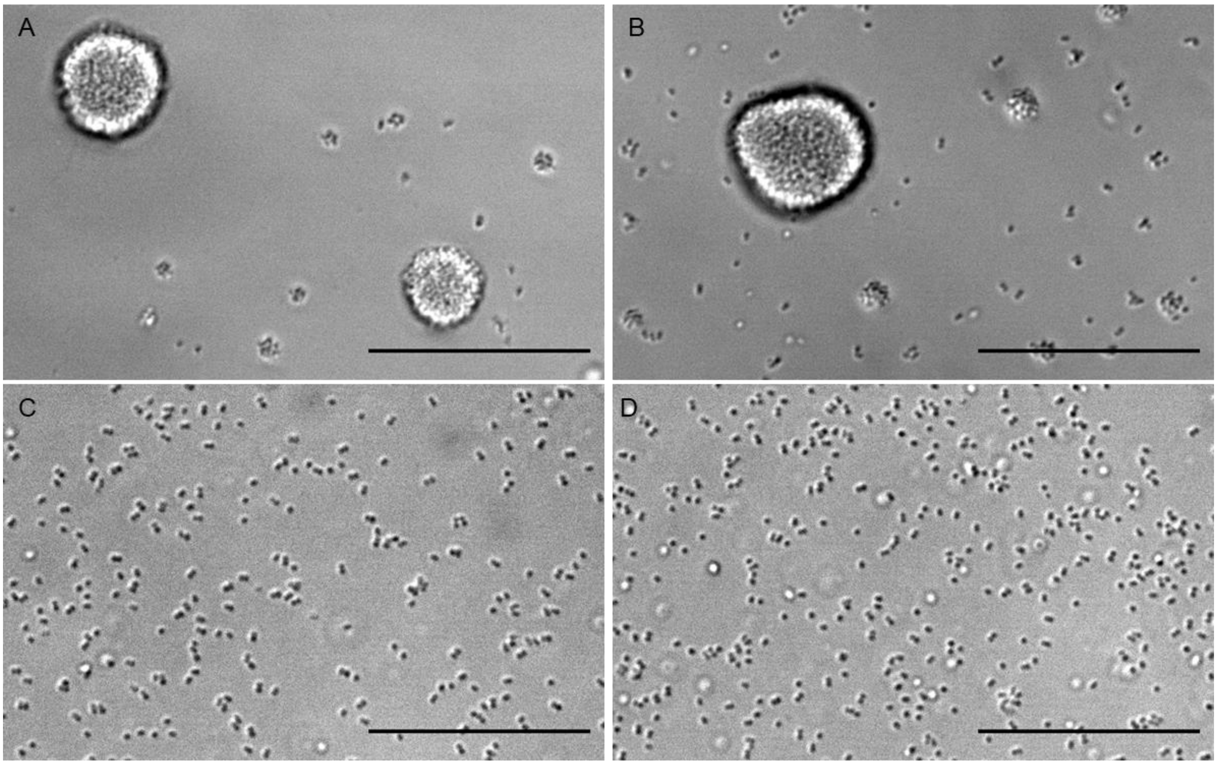

Figure x. Pilin glycosylation does not impact the colony phenotype of PilE variants. Representative images of PilE variants in a Δ*pglF* background. A) wt* Δ*pglF* (Ng156), B) wt_{pilE24} Δ*pglF* (Ng312), C) wt_{pilE17} Δ*pglF* (Ng311), D) wt_{pilE32} Δ*pglF* (Ng313). Scale bar: 50 μm.

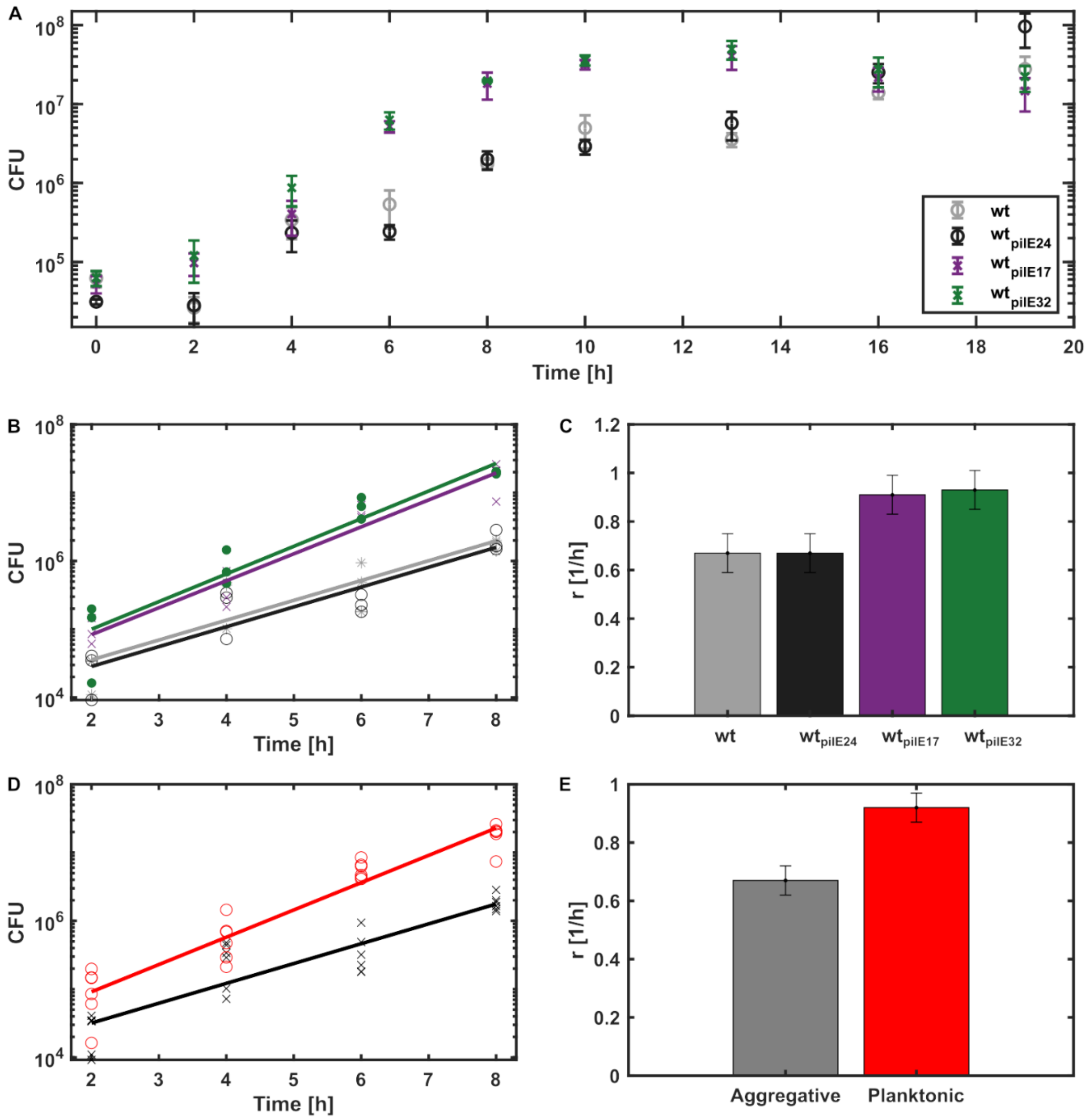

Figure xi. Growth rates for strains wt* (Ng150), wt$_{\textit{pilE24}}$ (Ng242), wt$_{\textit{pilE17}}$ (Ng240), wt$_{\textit{pilE32}}$ (Ng230). A) Growth curves for all *pilE* variants from counting colony forming units. N=3-4. Error bars: standard errors. B), D) Linear regression fits to logarithmic data of CFU counts of each strain or pooled data regarding the lifestyle: planktonic or aggregating. C), D) Growth rates determined from fits in B) and D), respectively. Error bars: errors of the fits. Anova-test of the linear regression model indicated no significant difference between the growth rates of the individual strains with $p_{\text{wt-wt}\textit{pilE24}} = 0.995$, $p_{\text{wt-wt}\textit{pilE17}} = 0.071$, $p_{\text{wt-wt}\textit{pilE32}} = 0.074$ but significant differences for pooled data $p_{\text{aggreg.-planktonic}} = 0.0074$.

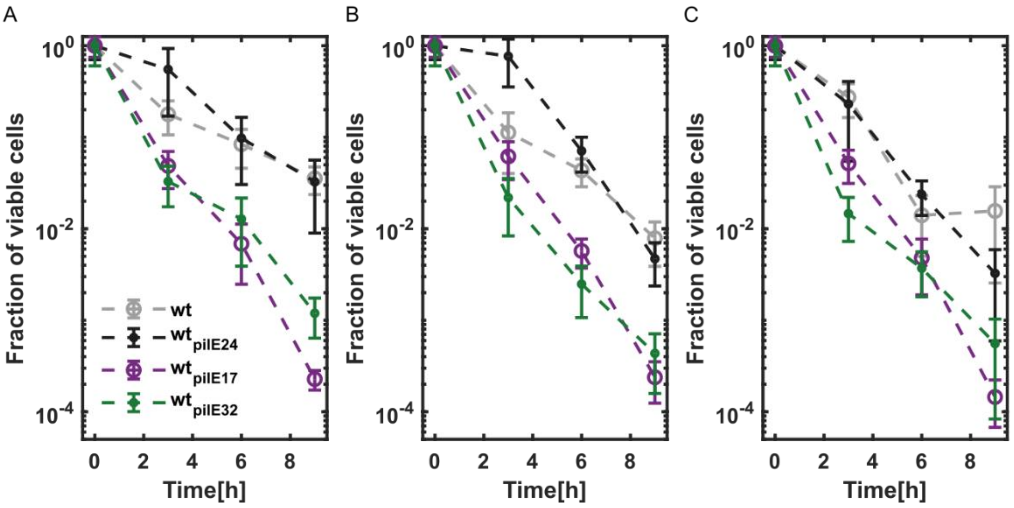

Figure xii. Survival assay with different concentrations of ceftriaxone after 10 h of growth. Killing kinetics of all variants, grey: wt*, dark grey: wt$_{pilE24}$, purple: wt$_{pilE17}$, and green: wt$_{pilE32}$, for A) 300x MIC, combined p-values (see Methods): $p_{wt\text{-}wt\mathit{pilE24}} = 0.63$, $p_{wt\text{-}wt\mathit{pilE17}} = 0.00029$, $p_{wt\text{-}wt\mathit{pilE32}} = 0.0011$; B) 600x MIC, $p_{wt\text{-}wt\mathit{pilE24}} = 0.35$, $p_{wt\text{-}wt\mathit{pilE17}} = 8.8\times10^{-5}$, $p_{wt\text{-}wt\mathit{pilE32}} = 0.00035$; C) 1200x MIC. $p_{wt\text{-}wt\mathit{pilE24}} = 0.99$, $p_{wt\text{-}wt\mathit{pilE17}} = 0.0038$, $p_{wt\text{-}wt\mathit{pilE32}} = 0.0013$. Error bars: standard errors over 3-4 independent experiments. Shown are mean and standard error over 3-4 biological culture replicates. The data underlying this figure can be found in S1 Data.

Supplementary Tables

**strain**	**relevant genotype**	**reference**	**strain data base entry**
N. gonorrhoeae NG32	-	clinical isolate	Ngc001
N. gonorrhoeae NG17	-	clinical isolate	Ngc004
N. gonorrhoeae NG24	-	clinical isolate	Ngc007
ΔG4 (wt)*	*G4::apraR*	[26]	Ng150
T126C step1	*G4::apraR, pilE::pilE*^{T126C} *ermC rpsL*$_{s}$	[19]	Ng225
ΔcomE$_{234}$*ΔpilV*	*comE4::Kan, comE3::Clm, comE2::Erm, recA6ind(tetM) pilVfs*	[63]	Ng052
wt$_{pilE32}$ step1	*G4::apraR, pilE::pilE*$_{clinicalisolateNG32}$ *ermC rpsL*$_{s}$	this study	Ng229
wt$_{pilE32}$	*G4::apraR, pilE*$_{clinicalisolateNG32}$	this study	Ng230
wt$_{pilE17}$ step1	*G4::apraR, pilE::pilE*$_{clinicalisolateNG17}$ *ermC rpsL*$_{s}$	this study	Ng239
wt$_{pilE17}$	*G4::apraR, pilE*$_{clinicalisolateNG17}$	this study	Ng240
wt$_{pilE24}$ step1	*G4::apraR, pilE::pilE*$_{clinicalisolateNG24}$ *ermC rpsL*$_{s}$	this study	Ng241
wt$_{pilE24}$	*G4::apraR, pilE*$_{clinicalisolateNG24}$	this study	Ng242
wt* $_{green}$	*G4::apraR, iga::P*$_{pilE}$*gfp-ermC*	[11]	Ng105
wt*$_{red}$	*G4::apraR, lctp:P*$_{pilE}$*mcherry aadA:aspC*	[9]	Ng170
wt* Δ*pglF*	*G4::apraR, pglF::P*$_{pilE}$*gfp-kanR*	[11]	Ng156
wt$_{pilE17green}$	*G4::apraR, pilE*$_{clinicalisolateNG17}$ *iga::P*$_{pilE}$*gfp-ermC*	this study	Ng308
wt$_{pilE24green}$	*G4::apraR, pilE::pilE*$_{clinicalisolateNG24}$ *iga::P*$_{pilE}$*gfp-ermC*	this study	Ng309
wt$_{pilE32green}$	*G4::apraR, pilE*$_{clinicalisolateNG32}$ *iga::P*$_{pilE}$*gfp-ermC*	this study	Ng310
wt$_{pilE17_T136}$ step1	*G4::apraR, pilE*$_{clinicalisolateNG17}$$^{K137-E160}$ *::pilE*$_{wt}$$^{T136-K160}$ *ermC rpsL*$_{s}$	this study	Ng292

wt$_{pilE17_T136}$	*G4::apraR*, $pilE_{clinicalisolateNG17}^{K137\text{-}E160}$ $::pilE_{wt}^{T136\text{-}K160}$	this study	Ng293
wt$_{pilE32_T136}$ step1	*G4::apraR*, $pilE_{clinicalisolateNG32}^{T135\text{-}P158}$ $::pilE_{wt}^{T136\text{-}K160}$ *ermC* $rpsL_{s}$	this study	Ng294
wt$_{pilE32_T136}$	*G4::apraR*, $pilE_{clinicalisolateNG32}^{T135\text{-}P158}$ $::pilE_{wt}^{T136\text{-}K160}$	this study	Ng295
wt$_{pilE17_K155}$ step1	*G4::apraR*, $pilE_{clinicalisolateNG17}^{E156\text{-}P158}$ $::pilE_{wt}^{K155\text{-}K160}$ *ermC* $rpsL_{s}$	this study	Ng304
wt$_{pilE17_K155}$	*G4::apraR*, $pilE_{clinicalisolateNG17}^{E156\text{-}P158}$ $::pilE_{wt}^{K155\text{-}K160}$	this study	Ng305
wt$_{pilE32_K155}$ step1	*G4::apraR*, $pilE_{clinicalisolateNG32}^{L157\text{-}P158}$ $::pilE_{wt}^{K155\text{-}K160}$ *ermC* $rpsL_{s}$	this study	Ng306
wt$_{pilE32_K155}$	*G4::apraR*, $pilE_{clinicalisolateNG32}^{L157\text{-}P158}$ $::pilE_{wt}^{K155\text{-}K160}$	this study	Ng307
$\Delta pilE_{green}$	*pilE::gfp-kanR*	[61,11]	Ng081
wt$_{pilE17}$ Δ*pglF*	$pglF::P_{pilE}gfp\text{-}kanR$	this study	Ng311
wt$_{pilE24}$ Δ*pglF*	$pglF::P_{pilE}gfp\text{-}kanR$	this study	Ng312
wt$_{pilE32}$ Δ*pglF*	$pglF::P_{pilE}gfp\text{-}kanR$	this study	Ng313
Δ*pilE*	*pilE::kanR*	this study	Ng253

Table i Strains used in this study.

Region	PilE variant	Query Cover [%]	Identity [%]	e-value	length
complete	NG17	98.0	80.6	4e-85	167
	NG32	96.0	86.6	5e-95	165
	NG24	99.0	90.0	3e-104	171
without conserved	NG17	94.0	73.6	5e-48	115
	NG32	94.0	80.4	3e-57	113
	NG24	99.0	85.6	2e-66	119
Semi-variable	NG17	100.0	76.1	1e-43	67
	NG32	100.0	80.6	2e-37	66
	NG24	100.0	89.6	2e-36	67
cys1	NG17	100.0	84.6	3e-13	13
	NG32	100.0	100.0	2e-15	13
	NG24	100.0	100.0	2e-15	13
Hypervariable loop	NG17	94.0	57.9	5e-06	20
	NG32	100.0	59.1	1e-09	22
	NG24	100.0	56.5	2e-08	23
cys2	NG17	100.0	100.0	5e-12	10
	NG32	100.0	100.0	5e-12	10
	NG24	100.0	100.0	5e-12	10
Hypervariable tail	NG17	-	-	-	5
	NG32	-	-	-	2
	NG24	83.0	100.0	5e-05	6

Table ii. Amino acid sequence identities of complete and partial regions of *pilE* compared to the MS11 *pilE* amino acid sequence according to Fig. 1. The amino acid sequences were compared using BLAST [70].

Primer	5' →3'
sk5	CCGCTCGAGCGGTTCCGACCCAATCAACACACC
sk32	GGGCCTTGAAGCGCAATCGATATA
sk45	CAACCCTTAAAGGAAAAACCATGCAATAC
sk46	TTGTATTCAGACGGCCAGTGACTTTTTGAAGGGTATTCATAAAATTACTCCTAATTG
sk47	TAATTTTATGAATACCCTTCAAAAAGTCACTGGCCGTCTGAATACAAC
sk48	GGGTAAAGCTTAGAAAAACTCATCGAGCATCAAATGAAAC
sk49	AATAAATTGCAGTTTCATTTGATGCTCGATGAGTTTTTCTAAGCTTTACCCTTATCGAGCT GATGATTGT
sk50	CATTTTCGGCTCCTTATTCGGTTTGAC
sk129	TTCCGACCCAATCAACACA
sk131	TTTAAGGCCTAATTTGCCTCATTTGGCATCAGATGCCTTAT
sk132	ATCTGATGCCAAATGAGGCAAATTAGGCCTTAAATTTTA
sk158	GTATCGGCAATGACGGTTCG
sk159	GAGTTGTCTCTGACACATTCTGG
sk159	GAGTTGTCTCTGACACATTCTGG
sk160	GAAGGGTATTCATAAAATTACTCCTAATTGAAAGGGG
sk161	CAATTAGGAGTAATTTTATGAATACCCTTCAAAAAGGCTTTACCC
sk162	GGTGATGAAGCACCGGGTCACGGCAGGTCACGG
sk163	TGACCCGGTGCTTCATCACC
sk164	TGAGGCAAATTAGGCCTTAAATTTTAAATAAATC
sk165	GATTTATTTAAAATTTAAGGCCTAATTTGCCTCACGGCAGGTCACGG
sk167	GGTGATGAAGCACCGGGTCAGGCGGCATCAGATGC
sk168	GATTTATTTAAAATTTAAGGCCTAATTTGCCTCAGGCGGCATCAGATG
sk175	GGTGATGAAGCACCGGGTCATTCGGCAGTTGATTCATCACG
sk176	GATTTATTTAAAATTTAAGGCCTAATTTGCCTCATTCGGCAGTTGATTCATCACG
sk384	ACCACTGCTGTGAGCGC
sk385	GCGGTGACGGCGTCGTCGGCGTCTTTGGCGTTGCG
sk386	CAAAGACGCCGACGACGCCGTCAC
sk388	GCCGTCGGCGGTAACGTCGTCGTTGGCTTTGTC
sk389	GCCAACGACGACGTTACCGCCGACGGCAAC
sk390	GTTACCAAACTCGACGGCACG
sk391	GTCATTTGGCATCAGATGCCTTATCACGGCAGGTTGACGG
sk392	CGTCAACCTGCCGTGATAAGGCATCTGATGCCAAATGACC
sk393	CATTTGGCATCAGATGCCTTGTCACGGCAGGTTGACGG
sk394	CGTCAACCTGCCGTGACAAGGCATCTGATGCCAAATGACC
sk395	TTTAAGGCCTAATTTGCCTCATTTGGCATCAGATGCCTTGTC

Table iii: Primers used in this study.
